# Assemblage of indigenous arbuscular mycorrhizal fungi and green waste compost enhance drought stress tolerance in carob (*Ceratonia siliqua* L.) trees

**DOI:** 10.1038/s41598-021-02018-3

**Published:** 2021-11-24

**Authors:** Abderrahim Boutasknit, Marouane Baslam, Mohamed Ait-El-Mokhtar, Mohamed Anli, Raja Ben-Laouane, Youssef Ait-Rahou, Toshiaki Mitsui, Allal Douira, Cherkaoui El Modafar, Said Wahbi, Abdelilah Meddich

**Affiliations:** 1 Laboratoire Agro-Alimentaire, Biotechnologies et Valorisation des Bioressources Végétales, Faculté des Sciences Semlalia, Cadi Ayyad University, BP: 2390, 40 000 Marrakesh, Morocco; 2 Department of Applied Biological Chemistry, Faculty of Agriculture, Niigata University, Niigata, 950-2181 Japan; 3 Laboratoire de Botanique et de Protection des Plantes, Faculté des Sciences, Ibn Tofail University, BP: 133, 14000 Kénitra, Morocco; 4 Laboratoire de Biotechnologie et Bio-Ingénierie Moléculaire, Faculté des Sciences et Techniques Guéliz, Cadi Ayyad University, BP: 2390, 40 000 Marrakech, Morocco

**Keywords:** Arbuscular mycorrhiza, Drought

## Abstract

In the current study, an eco-friendly management technology to improve young carob (*Ceratonia siliqua* L.) tree tolerance to water deficit was set up by using single or combined treatments of arbuscular mycorrhizal fungi (AMF) and/or compost (C). Two groups of young carob have been installed: (i) carob cultivated under well-watered conditions (WW; 70% field capacity (FC)) and (ii) where the plants were drought-stressed (DS; 35% FC) during 2, 4, 6, and 8 months. The effect of used biofertilizers on the course of growth, physiological (photosynthetic traits, water status, osmolytes, and mineral content), and biochemical (hydrogen peroxide (H_2_O_2_), oxidative damage to lipids (malondialdehyde (MDA), and membrane stability (MS)) traits in response to short- and long-term droughts were assessed. The dual application of AMF and C (C + AMF) boosted growth, physiological and biochemical parameters, and nutrient uptake in carob under WW and DS. After eight months, C + AMF significantly enhanced stomatal conductance by 20%, maximum photochemical efficiency of PSII by 7%, leaf water potential by 23%, chlorophyll and carotenoid by 40%, plant uptake of mineral nutrients (P by 75%, N by 46%, K^+^ by 35%, and Ca^2+^ by 40%), concentrations of soluble sugar by 40%, and protein content by 44% than controls under DS conditions. Notably, C + AMF reduced the accumulation of H_2_O_2_ and MDA content to a greater degree and increased MS. In contrast, enzyme activities (superoxide dismutase, catalase, peroxidase, and polyphenoloxidase) significantly increased in C + AMF plants under DS. Overall, our findings suggest that the pairing of C + AMF can mediate superior drought tolerance in young carob trees by increasing leaf stomatal conductance, cellular water content, higher solute concentration, and defense response against oxidative damage during the prolonged period of DS.

## Introduction

Mediterranean ecosystems are characterized recently by scarce and irregular rainfall year-to-year and prolonged periods of drought—usually lasting for several months—, which alter them and cause several negative impacts^1^. Drought is becoming one of the most critical environmental stresses outside of plants’ physiological limits and is causing a substantial decline in crop productivity, particularly in arid and semi-arid regions^2,3^. This constraint affects plants' morphological, physiological, and nutritional traits, including water content, leaf water potential, photosynthetic pigment, stomatal conductance, photochemical efficiency of PSII, and mineral nutrients uptake^4^. Drought also affects membrane stability, leading to lipid and protein damage due to reactive oxygen species (ROS)^5^. Nevertheless, drought-inducing detrimental effects on growth and crop productivity depend on several factors: the level of stress experienced, stress length, the rainfall pattern during the growing season, and the soil physical and chemical properties^6^. Recent widespread of Mediterranean forests and scrublands mortality associated with drought and/or temperature stress will only worsen over the next few decades^7^, thereby potentially affecting biodiversity, risk of wildfire, nutrient cycling, hydrology, land–atmosphere interactions, and resilience of ecosystems services supplied to humans on a regional to global scale^8^. The substitution of some species by others more resilient, improving our understanding of the plant capacity to acclimate/adapt to ‘new’ conditions and emergent occurring phenomena—e.g. forest fires and avalanches—and successful forest management strategies should be prioritized to minimize adverse effects.

The carob (*Ceratonia siliqua* L.) is considered an essential component of the arboreal flora of the Mediterranean basin owing to its drought tolerance capacity and adaptation to degraded and impoverished plants nutrient soils^9,10^. The carob tree was widely known as the ‘black gold’ due to the numerous health benefits and nutritional value—rich in dietary fibres, minerals, and low in lipids, has abundance in D-pinitol and antioxidants, such as polyphenols and tannins—, thermo-tolerant volatile organic compounds aroma, and livestock feed in agroforestry systems^11–13^. Carob leaves and pods have numerous functional, pharmaceutical, and cosmetic properties and applications. Recent studies have shown that this plant extracts exhibit antioxidant, antidiarrheal, antibacterial, antifungal, anti-inflammatory, antidiabetic activities, and hepatoprotective and antiproliferative effects^14,15^. It was reported that the carob tree could be used as a neurodegenerative disorders therapy^14^ and in the prevention of free radical related diseases as a natural food supplement^16^. Moreover, the carob tree is generally used in biotechnological fields as a natural bioactive resource for the food industry. Indeed, the locust bean gum derived from the seed coat is used as a food additive (E410), having thickening, stabilizing, flavoring and emulsifying functions^17^. In addition, the seeds are also used in cosmetic industries (e.g. associated with its antioxidant/preservative function)^18^.

Traditionally, the carob is an indigenous drought- and temperature-tolerant tree, benefitting thereby the agricultural economy—136,540 tons per year worldwide—, however, the increase in labor cost and the era of ever-lower pricing in the international market gradually resulted in the abandonment of the crop^9,19^. The drought tolerance of carobs depends on morphological, physiological, and biochemical mechanisms that reduce stress^20,21^.

To combat climate change-induced drought in Mediterranean crops, management practices and strategies that allow plants to resist abiotic stresses are urgently required and should be exploited to improve agricultural production and protect crops and soil quality^22^. The long-term sustainable development of natural resource management is increasingly supported by integrating environmental health and economic profitability. The design of integrated biological agroecosystems based on soil nutrient cycling would help maintain an economical production system. Thus, the application of biofertilizers such as organic fertilizers (compost) and beneficial symbiotic microorganisms (i.e. arbuscular mycorrhizal fungi) has emerged as a potential solution to improve soil nutrients and water availability, productivity, and resistance of plants to harsh environmental conditions, particularly in forestry programs^23–26^. Recently, using organic compost as an alternative to chemical fertilizers has become a global consensus increasingly. It has been demonstrated to be efficient to improve the resilience, yield, and tolerance of plants to harsh conditions^26^. Compost application could enhance mineral nutrition, soil organic matter content, and soil properties such as water-holding capacity^27,28^. Previous studies have shown that the application of organic fertilizer in soil enhanced tolerance to environmental stresses through, among others, enhanced microbial activity and soil fungal to the bacterial ratio^29^. By contributing significantly to the acquisition of nutrient release of the compost through biochemical transformations, microorganisms play an essential role to ensure stable, safe and sustainable agricultural and biomass production^30^. The symbiotic association between arbuscular mycorrhizal fungi (AMF) and plant roots is one of the most known beneficial interactions in soil^31^. Thus, they are collecting growing interest as natural fertilizers.

AMF form intimate associations with about 80% of land plant species, with the fungus gaining photosynthate in return for supplying the host plant with mineral nutrients and water^32^. AMF increase plant drought resistance owing to the well-developed extra-radical mycorrhizal mycelia that proliferate in the bulk soil beyond the rhizosphere and increase the soil volume connected to the plant^31^. These mycelia's ability to enlarge the absorption range of roots accelerates the water and nutrients uptake, especially the P element, which moves 10 × faster in mycelium than in roots^33,34^. The mycorrhizal symbiosis can also help the host plants to improve their performance under water stress situations by increasing the stomatal conductance and photosynthesis^35^. AMF can mitigate the detrimental effect of drought stress by restraining reactive oxygen species (ROS) overproduction in plant cell^36,37^. Less accumulation of ROS in mycorrhizal plants is owing to the enhancement of non-enzymatic antioxidants (e.g. ascorbate and glutathione) concentrations and/or antioxidant enzyme (e.g. superoxide dismutase, catalase, peroxidases) activities^38^, and/or AMF-mediated ROS signaling, such as root H_2_O_2_ effluxes^39^. Under drought conditions, plants alter water relations by synthesizing osmolytes to maintain turgidity pressure and cellular functions necessary for the metabolic processes^40^. One of the crucial biotic soil factors determining the fungal inoculant's success is the indigenous mycorrhizal community. Indeed, different combinations of AMF taxa differentially interact and can exhibit functional complementarity^41,42^. Therefore, inoculation of compatible AMF—and selecting plant species—could be an effective method for re-vegetation in the arid area. However, the management practices that integrate a better compost application combined with soil microbes' beneficial roles remain poorly investigated ^24,43^, and the associated regulatory mechanisms remain mostly obscure. In the current study, we attempted to (1) investigate the functionality of compost amendment and/or AMF inoculation on young carob tree to improve its drought tolerance as a high priority for the ideal plant for afforestation, and (2) assess the development of growth, physiological, and biochemical responses involved in carob under the implementation of eco-friendly cultivation practices and subjected to long-term drought. The obtained results will provide a deeper understanding of the mechanisms of carob tolerance to long-term lack of water and pave the way for the theoretical basis for applying emerging technologies and management of crop stress tolerance in the ecologic restoration of the arid forest. Therefore, we hypothesized (1) that the drought-induced reduction of carob trees will be mitigated by native AMF and/or compost. Moreover, we expected (2) that interactive effects of AMF and compost could induce greater growth and stress tolerance of carob through improved photosynthetic apparatus, alleviated oxidative damage, and enhanced ROS generation and scavenging via its modulatory role in the activities of antioxidant enzymes under stressful condition.

## Results

### Mycorrhizal status and carob growth

**Figure 1:**
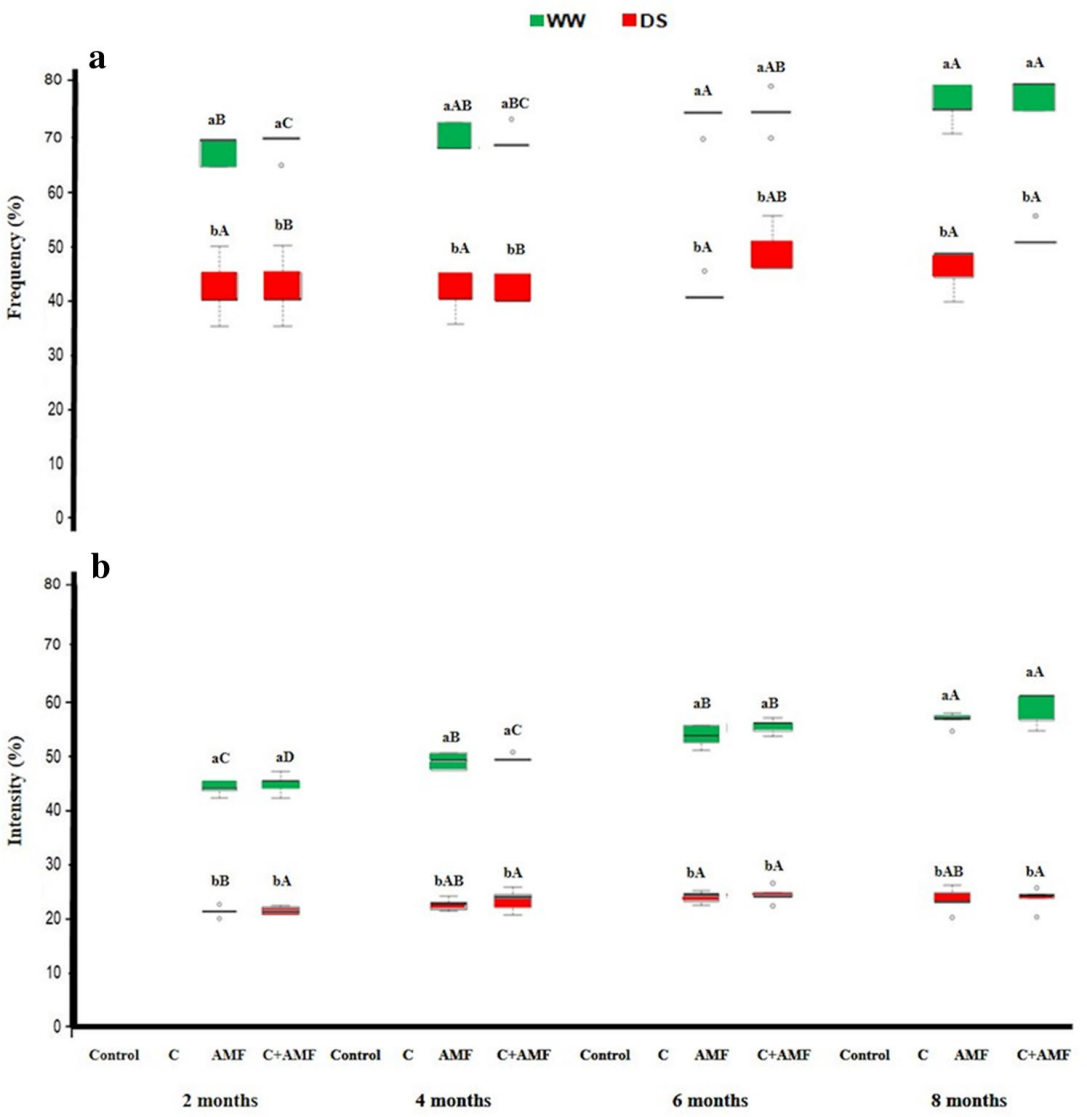
Effects of water regimes (WW: well-watered; open bars and DS: drought stress; filled bars) on **(a)** AMF infection frequency and (**b**) intensity in control carob plants and plants amended with compost (C) and/or assemblage of indigenous arbuscular mycorrhizal fungi (AMF). Data are mean ± SE (n = 5). Means followed by the same letters within the same time point are not significantly different at *P* < 0.05 (Tukey’s HSD). The upper case represents the differences within WW or DS water regimes, and the lower case represents the differences within a one-time point.

There was no AM colonization detectable in untreated plants (Fig. 1). The frequency and intensity of mycorrhization of young carob tree roots were significantly affected by AMF, compost, drought, and their interactions (Table 1). Plants inoculated with AMF consortium alone or in combination with compost (AMF and C + AMF) yielded higher frequency (F% > 67%) and intensity (I% > 48%) of mycorrhization percentages under WW regime than DS conditions. Indeed, under WW, the intensity of infection was ca. 2 × higher in C + AMF compared to those inoculated and amended plants subjected to DS along with the carob culture. Under DS, mycorrhizal frequency remained stable throughout the 2-, 4-, 6-, and 8-months drought periods in AMF inoculated plants, while the addition of compost increased + 20% the F% in the 2–8 period (Fig. 1). Under DS, mycorrhizal intensity recorded no significant development for all treatments over time except for AMF treatment, which significantly increased in the 2–6 period.

**Table 1:** ANOVA test results for independent variables; (A) AMF, (C) compost, (D) drought, and (T) timing of the measured parameters in carob.

Parameters	A	C	D	T	A × C	A × D	A × T	C × D	C × T	D × T	A × C × D	A × C × T	A × D × T	C × D × T	A × C × D × T
Frequency of mycorrhization (F%)	***	***	***	***	***	***	***	**	*	NS	**	*	*	NS	NS
Intensity of mycorrhization (I%)	***	*	***	***	*	***	***	NS	NS	***	NS	NS	NS	NS	NS
Shoot Height (SH)	***	***	***	***	***	*	***	*	***	***	NS	*	*	NS	*
Root Length (RL)	***	***	***	***	NS	NS	*	NS	NS	***	NS	NS	NS	NS	NS
Shoot Dry Matter (SDM)	***	***	***	***	*	NS	***	NS	***	***	***	***	***	NS	**
Root Dry Matter (RDM)	***	***	***	***	NS	**	***	***	NS	***	**	*	*	NS	*
Leaf water potential (Ψ_Leaf_)	***	***	***	***	***	***	NS	***	NS	***	NS	*	*	NS	NS
Relative Water Content (RWC)	***	***	***	***	***	***	NS	***	NS	***	NS	*	*	NS	NS
Stomatal conductance (g_s_)	***	***	***	***	**	*	*	***	*	**	*	NS	NS	*	*
Chlorophyll fluorescence (F_v_/F_m_)	***	***	***	NS	***	NS	NS	NS	NS	NS	NS	NS	NS	NS	NS
Chlorophyll a (Chl a)	***	***	***	***	**	*	NS	***	NS	***	***	**	**	NS	***
Chlorophyll b (Chl b)	***	***	***	***	***	***	NS	**	*	***	***	*	**	*	NS
Total Chlorophyll (Chl T)	***	***	***	***	***	***	NS	NS	*	***	***	*	**	*	NS
Carotenoid (Car)	***	***	***	***	***	***	***	***	***	NS	*	***	***	NS	***
Total soluble sugar (TSS)	***	***	***	***	NS	*	***	NS	***	**	NS	***	***	***	***
Protein	***	***	***	***	NS	**	***	NS	NS	***	NS	NS	NS	NS	NS
Membrane stability (MS)	***	***	***	***	***	***	NS	NS	NS	***	*	**	**	NS	NS
Hydrogen peroxide (H_2_O_2_)	***	***	***	***	***	NS	***	**	**	***	NS	***	***	***	***
Malondialdehyde (MDA)	***	***	***	***	***	***	***	***	NS	**	NS	***	***	***	NS
Nitrogen (N)	***	***	***	***	***	***	**	***	NS	NS	*	NS	NS	NS	*
Phosphorus (P)	***	***	***	***	***	***	***	NS	***	***	***	***	***	***	***
Potassium (K)	***	***	***	***	***	***	***	***	***	***	***	***	***	***	***
Calcium (Ca)	***	***	***	***	***	***	***	***	***	***	***	***	***	***	***
Superoxide dismutase (SOD)	***	***	***	***	***	***	***	*	***	***	***	***	**	**	***
Catalase (CAT)	***	***	***	***	***	***	***	***	***	***	NS	***	***	***	***
Peroxidase (POX)	***	***	***	***	***	***	NS	***	NS	*	***	*	***	**	NS
Polyphenoloxidase (PPO)	***	***	***	***	**	*	***	NS	***	NS	NS	NS	NS	NS	NS

NS, not significant, *P < 0.05, **P < 0.01, ***P < 0.001.

The growth of carob seedlings significantly (*P* < 0.001) varied depending on the water regime, biofertilizers application, and length of treatments (Table 2). Biofertilizers benefited the shoot and root dry weights, plant height, and root length in young carob under WW and DS conditions. At short-term, 2-month drought stress, the young control carob showed a significant decrease (*P* < 0.001) on growth parameters including shoot height (SH) by −19.4%, root length (RL) by −17.9%, shoot dry matter (SDM) by −41.4% and root DM (RDM) by −32% than WW regime. This decrease has been exacerbated after eight months of stress to reach ca. −58% for SDM and −41% for RDM than WW plants. The application of AMF and/or organic amendments allowed the growth and development of carob in drought conditions. Following a 6-month drought, the application of C and AMF alone and their combination C + AMF significantly improved SH and RL to a greater extent than in non-inoculated and non-amended plants. Moreover, C + AMF showed the highest SDM and RDM values under WW and DS compared to untreated plants. The carob treated with C + AMF under DS was significantly (*P* < 0.05) taller with higher biomass than controls. This highest gain in biomass accumulation under DS was more important in shoots (59%, 65%, 70% and 92%) than in roots (12%, 12%, 19%, and 21%) at 2, 4, 6, and 8 months, respectively compared to WW. Under DS conditions, all treatments showed a great improvement throughout the 2-, 4-, 6-, and 8-months drought period. The effect of interactions among all factors (AMF x compost x drought x timing) was significant (*P* < 0.05) for SH, SDM, and RDM (Table 1).

**Table 2:** Growth parameters (SH: Shoot Height, RL: Root Length, SDM: Shoot Dry Matter, RDM: Root Dry matter) in carob under different water status (WW: well-watered, DS: drought stress) and/or biofertilizers (C: compost, AMF: arbuscular mycorrhizal fungi, C + AMF: compost and AMF consortium combination) after 2, 4, 6, and 8 months of treatments’ application.

		SH (cm)		RL (cm)		SDM (g)		RDM (g)	
		WW	DS	WW	DS	WW	DS	WW	DS
2 months	Control	24.2 ± 0.7 aD	19.5 ± 0.8 bC	19.6 ± 1.8 a-dB	16.1 ± 1.3 eC	2.9 ± 0.5 a-cD	1.7 ± 0.3 dD	2.5 ± 0.6 bcB	1.7 ± 0.2 dB
	C	25.2 ± 1.0 aC	20.6 ± 1.0 bC	20.2 ± 1.3 a-cC	17.4 ± 0.8 deC	3.2 ± 0.5 abD	2.3 ± 0.4 cdD	3.8 ± 0.1 aC	1.8 ± 0.2 cdB
	AMF	24.9 ± 1.0 aD	20.1 ± 0.8 aD	21.0 ± 1.5 abC	17.9 ± 1.3 cdeC	3.6 ± 0.2 aD	2.6 ± 0.5 bcD	2.9 ± 0.5 abC	1.6 ± 0.4 dB
	C + AMF	24.8 ± 1.4 aC	20.2 ± 0.7 bC	22.2 ± 1.0 aC	19.1 ± 1.0 b-dC	3.7 ± 0.3 aD	2.8 ± 0.2 bcD	2.8 ± 0.6 abA	1.9 ± 0.2 cdC
4 months	Control	32.7 ± 0.7 bC	23.9 ± 0.6 dB	24.4 ± 1.3 aA	19.3 ± 1.0 cAB	5.0 ± 0.2 bC	2.5 ± 0.2 eC	3.1 ± 0.1 bB	1.9 ± 0.2 cB
	C	33.8 ± 1.2 abB	25.7 ± 1.4 cdB	25.5 ± 0.9 aB	20.8 ± 1.4 bcB	5.1 ± 0.5 bC	3.4 ± 0.2 dC	4.1 ± 0.2 aB	1.8 ± 0.2 cB
	AMF	34.8 ± 1.0 aC	26.1 ± 0.5 cC	25.4 ± 0.4 aB	21.2 ± 0.4 bcB	5.1 ± 0.2 bC	3.3 ± 0.2 dC	4.0 ± 0.1 aB	1.9 ± 0.2 cB
	C + AMF	33.3 ± 0.8 abB	25.7 ± 0.9 cdB	26.3 ± 1.6 aB	22.1 ± 1.6 bB	6.4 ± 0.2 aC	4.2 ± 0.2 cC	3.9 ± 0.5 aA	2.1 ± 0.5 cC
6 months	Control	47.3 ± 3.6 bB	31.2 ± 0.6 dA	25.8 ± 0.5 cA	20.0 ± 0.5 fAB	8.0 ± 0.7 bB	3.5 ± 0.3 dB	3.8 ± 0.4 bA	2.3 ± 0.2 cAB
	C	52.3 ± 0.6 aA	35.8 ± 1.1 cA	26.7 ± 0.9 bcAB	21.5 ± 0.9 efAB	9.0 ± 0.6 bB	5.6 ± 0.3 cB	4.8 ± 0.1 aA	2.4 ± 0.2 cA
	AMF	53.1 ± 0.6 aB	37.5 ± 1.1 cB	29.1 ± 0.9 aA	23.1 ± 0.9 deAB	8.6 ± 0.6 bB	5.4 ± 0.3 cB	4.9 ± 0.1 aA	2.6 ± 0.2 cA
	C + AMF	53.0 ± 2.9 aA	38.4 ± 0.9 cA	29.0 ± 2.0 abA	24.4 ± 2 cdA	11.2 ± 0.8 aB	6.0 ± 0.2 cB	5.4 ± 0.9 aB	2.7 ± 0.4 cB
8 months	Control	52.4 ± 0.7 bA	30.4 ± 0.8 eA	26.0 ± 0.2 bcA	21.1 ± 0.9 dA	10.7 ± 0.4 cA	4.5 ± 0.3 fA	4.4 ± 0.3 cA	2.6 ± 0.5 dA
	C	54.6 ± 1.1 abA	36.2 ± 0.7 dA	28.0 ± 1.1 abA	23.7 ± 1.9 cA	13.4 ± 1.1 bA	7.1 ± 0.4 eA	5.0 ± 0.4 bcA	2.6 ± 0.2 dA
	AMF	55.5 ± 1.0 aA	39.2 ± 0.7 cA	29.7 ± 0.9 aA	23.9 ± 1.2 cA	13.7 ± 0.7 bA	7.9 ± 0.4 deA	5.3 ± 0.6 bA	2.9 ± 0.4 dA
	C + AMF	56.1 ± 1.8 aA	39.6 ± 1.9 cA	29.9 ± 0.7 aA	25.5 ± 1.5 cA	15.2 ± 0.3 aA	8.7 ± 0.4 dA	6.5 ± 0.4 aB	3.2 ± 0.1 dA

Means (± standard error) within the same parameter, followed by different letters, are significantly different among treatments at P ≤ 0.05. The upper case represents the differences within WW or DS water regime, and the lower case represents the differences within a one-time point.

### Water relations and mineral uptake efficiency

**Figure 2:**
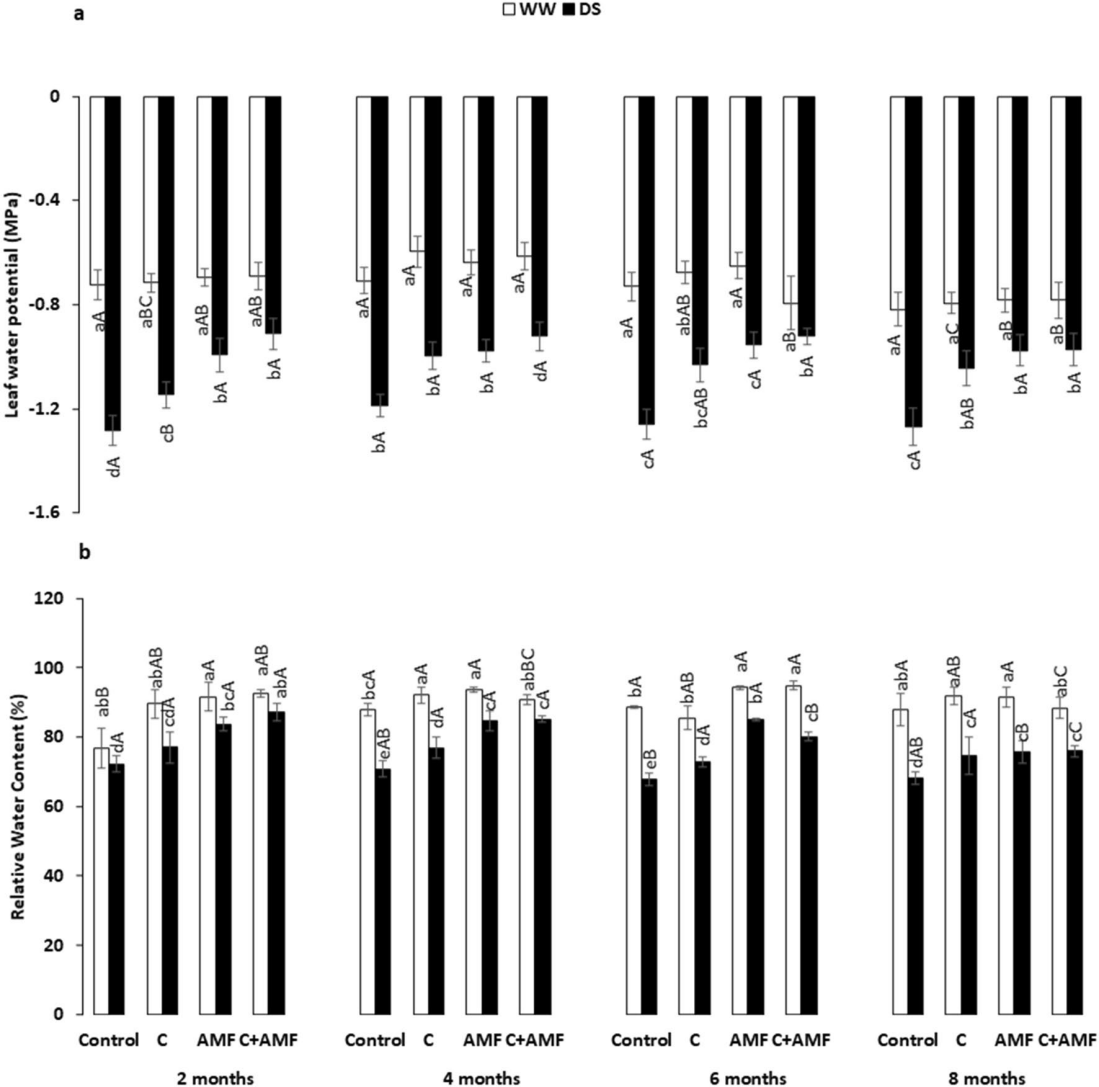
Influence of water regimes (WW: well-watered; open bars and DS: drought stress; filled bars) on (**a**) leaf water potential (Ψ_Leaf_) and (**b**) relative water content (RWC) in control carob plants and plants amended with compost (C) and/or assemblage of indigenous arbuscular mycorrhizal fungi (AMF). Data are mean ± SE (n = 5). Means followed by the same letters within the same time point are not significantly different at *P* < 0.05 (Tukey’s HSD). The upper case represents the differences within WW or DS water regimes, and the lower case represents the differences within a one-time point.

The leaf water potential (Ψ_Leaf_) (Fig. 2a) and relative water content (RWC) (Fig. 2b) significantly (*P* < 0.05) decreased with the severity of drought in untreated plants. Under WW, there was no significant effect of compost and/or AMF treatments on Ψ_Leaf_, while both treatments single or combined resulted in higher RWC in the short term. Under DS, in contrast, both treatments' effect was mainly significant than untreated plants, with higher values being observed in C + AMF treated plants. The dual application C + AMF increased Ψ_Leaf_ by ca. 30, 22, 27 and 23% and RWC by ca. 21, 20, 18 and 11% after 2, 4, 6 and 8 months, respectively, compared to the control plants under DS conditions. There was a significant interaction (*P* < 0.05) among the three main factors AMF x drought x timing on Ψ_Leaf_ and RWC (Table 1).

Overall, foliar nutrients concentrations were more significant in young carob inoculated with the AMF assemblage and/or amended with compost, independently of the water regime and the observation time (Tables 1 and 3). Indeed, from short-term, AMF and C + AMF treated plants displayed the highest foliar P, N, K, and Ca concentrations under DS (Table 3). These promotion effects induced by the biofertilizers were observed all along the experiment period. After 8 months, irrespective of water regimes, C + AMF showed a positive synergistic effect on foliar N, P, K, and Ca with the maximum increase (20, 65, 92 and 83%, respectively, under WW and 46, 75, 35 and 40%, respectively, under DS) compared with those untreated plants. The stressed plants did not show any significant variation for each treatment over time. The interactions among AMF, compost, drought, and timing on the nutrients content were significant at *P* < 0.05 (Table 1).

**Table 3:** Carob shoots mineral content subjected to different water status (WW: well-watered, DS: drought stress) and/or biofertilizers (C: compost, AMF: arbuscular mycorrhizal fungi, C + AMF: compost and AMF consortium combination) after 2, 4, 6, and 8 months of treatments’ application.

		P (mg g^−1^ DW)		N (mg g^−1^ DW)		K (mg g^−1^ DW)		Ca (mg g^−1^ DW)	
		WW	DS	WW	DS	WW	DS	WW	DS
2 months	Control	0.81 ± 0.01 eD	0.84 ± 0.02 deC	1.92 ± 0.09 aBC	1.14 ± 0.09 bC	1.43 ± 0.04 hD	2.40 ± 0.04 eC	4.15 ± 0.59 dD	3.99 ± 0.24 dD
	C	0.84 ± 0.02 deC	0.87 ± 0.01 dC	1.94 ± 0.33 aC	1.72 ± 0.18 aD	2.06 ± 0.06 gD	2.21 ± 0.02 fD	4.21 ± 0.44 dD	4.29 ± 0.32 dD
	AMF	0.87 ± 0.01 dD	1.18 ± 0.01 bC	1.94 ± 0.34 aC	1.85 ± 0.06 aD	2.61 ± 0.02 dD	3.70 ± 0.01 aD	6.86 ± 0.07 aD	6.02 ± 0.34 bcD
	C + AMF	0.99 ± 0.03 cD	1.50 ± 0.02 aD	2.00 ± 0.12 aC	1.92 ± 0.33 aD	2.86 ± 0.02 cB	3.31 ± 0.02 bD	6.92 ± 0.05 aD	6.38 ± 0.05 bA
4 months	Control	0.99 ± 0.01 eC	1.04 ± 0.03 dB	2.07 ± 0.10 bcB	1.44 ± 0.21 dB	1.82 ± 0.06 fC	3.41 ± 0.06 bBC	6.57 ± 0.61 cC	6.26 ± 0.19 deC
	C	1.02 ± 0.02 deB	1.20 ± 0.01 cB	2.23 ± 0.14 bB	1.98 ± 0.13 cC	2.38 ± 0.06 eC	2.76 ± 0.03 dC	7.43 ± 0.23 cC	6.56 ± 0.07 dC
	AMF	1.06 ± 0.02 dC	1.69 ± 0.02 abB	2.41 ± 0.06 aB	2.18 ± 0.10 bcC	2.95 ± 0.04 cC	3.97 ± 0.02 aC	8.31 ± 0.20 abC	7.50 ± 0.12 cC
	C + AMF	1.67 ± 0.04 abC	1.75 ± 0.03 aC	2.20 ± 0.14 bBC	2.35 ± 0.15 abBC	3.33 ± 0.01 bB	4.02 ± 0.02 aC	8.77 ± 0.23 aC	7.80 ± 0.35 bA
6 months	Control	1.11 ± 0.04 fB	1.20 ± 0.02 eA	2.17 ± 0.14 bAB	1.79 ± 0.15 cA	2.16 ± 0.02 hB	3.58 ± 0.11 deB	8.36 ± 0.30 fB	10.00 ± 0.12 eB
	C	1.42 ± 0.03 dA	1.48 ± 0.05 dA	2.31 ± 0.17 a-cAB	2.15 ± 0.03 bB	2.89 ± 0.02 gB	3.40 ± 0.05 fB	10.41 ± 0.33 dB	10.69 ± 0.16 dB
	AMF	1.72 ± 0.03 cB	1.81 ± 0.05 bA	2.61 ± 0.14 aAB	2.37 ± 0.03 bB	3.73 ± 0.06 cdB	4.29 ± 0.04 bB	12.86 ± 0.12 bB	11.84 ± 0.48 cB
	C + AMF	1.82 ± 0.01 bB	1.94 ± 0.03 aB	2.41 ± 0.10 abB	2.48 ± 0.03 aB	3.90 ± 0.03 cB	4.68 ± 0.02 aB	13.64 ± 0.27 aB	13.66 ± 0.22 aA
8 months	Control	1.22 ± 0.03 gA	1.21 ± 0.03 gA	2.39 ± 0.18 bA	1.90 ± 0.06 cA	2.29 ± 0.04 gA	3.89 ± 0.04 eA	10.41 ± 0.08 fA	13.40 ± 0.19 eA
	C	1.46 ± 0.02 fA	1.53 ± 0.02 eA	2.48 ± 0.06 bA	2.33 ± 0.13 bA	3.75 ± 0.03 fA	3.88 ± 0.02 eA	16.66 ± 0.12 dA	16.54 ± 0.20 dA
	AMF	1.92 ± 0.03 cA	1.87 ± 0.03 dA	2.76 ± 0.03 aA	2.73 ± 0.06 aA	4.31 ± 0.08 dA	4.90 ± 0.02 bA	17.63 ± 0.12 cA	17.47 ± 0.58 cA
	C + AMF	2.01 ± 0.03 bA	2.12 ± 0.06 aA	2.87 ± 0.13 aA	2.78 ± 0.14 aA	4.39 ± 0.02 cA	5.27 ± 0.05 aA	20.07 ± 0.19 aA	18.74 ± 0.17 bA

Means (± standard error) within the same parameter, followed by different letters, are significantly different among treatments at P ≤ 0.05. The upper case represents the differences within WW or DS water regime, and the lower case represents the differences within a one-time point.

### Stomatal conductance, efficiency of photosystem II, and photosynthetic pigments

**Figure 3:**
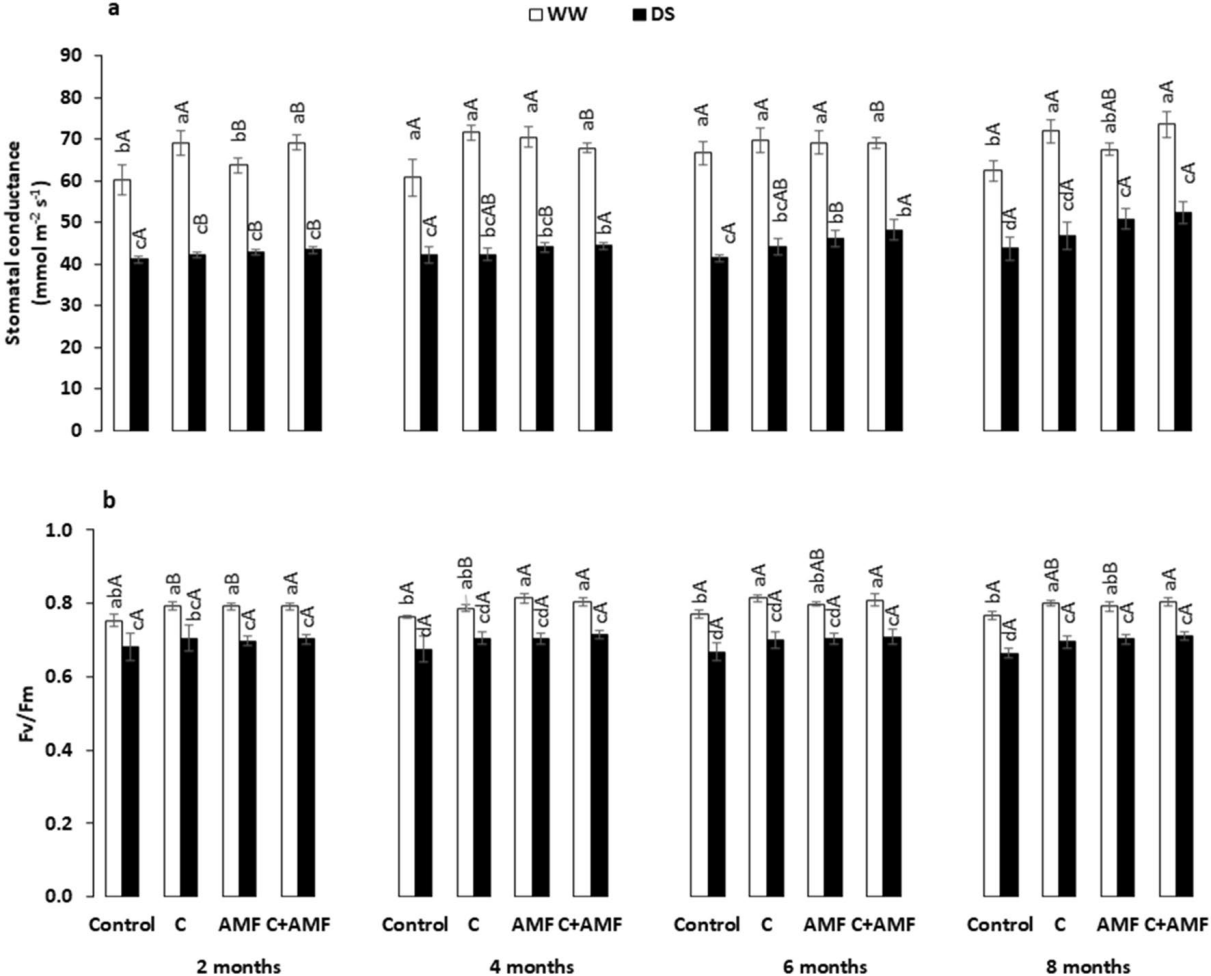
Influence of water regimes (WW: well-watered; open bars and DS: drought stress; filled bars) on (**a**) stomatal conductance; g_s_ and (**b**) chlorophyll fluorescence (F_v_/F_m_) in control carob plants and plants amended with compost (C) and/or assemblage of indigenous arbuscular mycorrhizal fungi (AMF). Data are mean ± SE (n = 5). Means followed by the same letters within the same time point are not significantly different at *P* < 0.05 (Tukey’s HSD). The upper case represents the differences within WW or DS water regimes, and the lower case represents the differences within a one-time point.

DS significantly decreased (*P* < 0.001) stomatal conductance (g_s_) (Fig. 3a) and quantum efficiency of photosystem II (F_v_/F_m_) (Fig. 3b), being reductions particularly pronounced in the absence of biofertilizers. These parameters increased significantly by C + AMF under DS conditions after 4, 6, and 8 months by ca. 5, 17, and 20%, respectively, for g_s_ and 6, 6, and 7%, respectively, for F_v_/F_m_ compared to the untreated carob. The interaction among AMF, compost, drought, and timing significantly affected g_s_ (*P* < 0.05).

**Figure 4:**
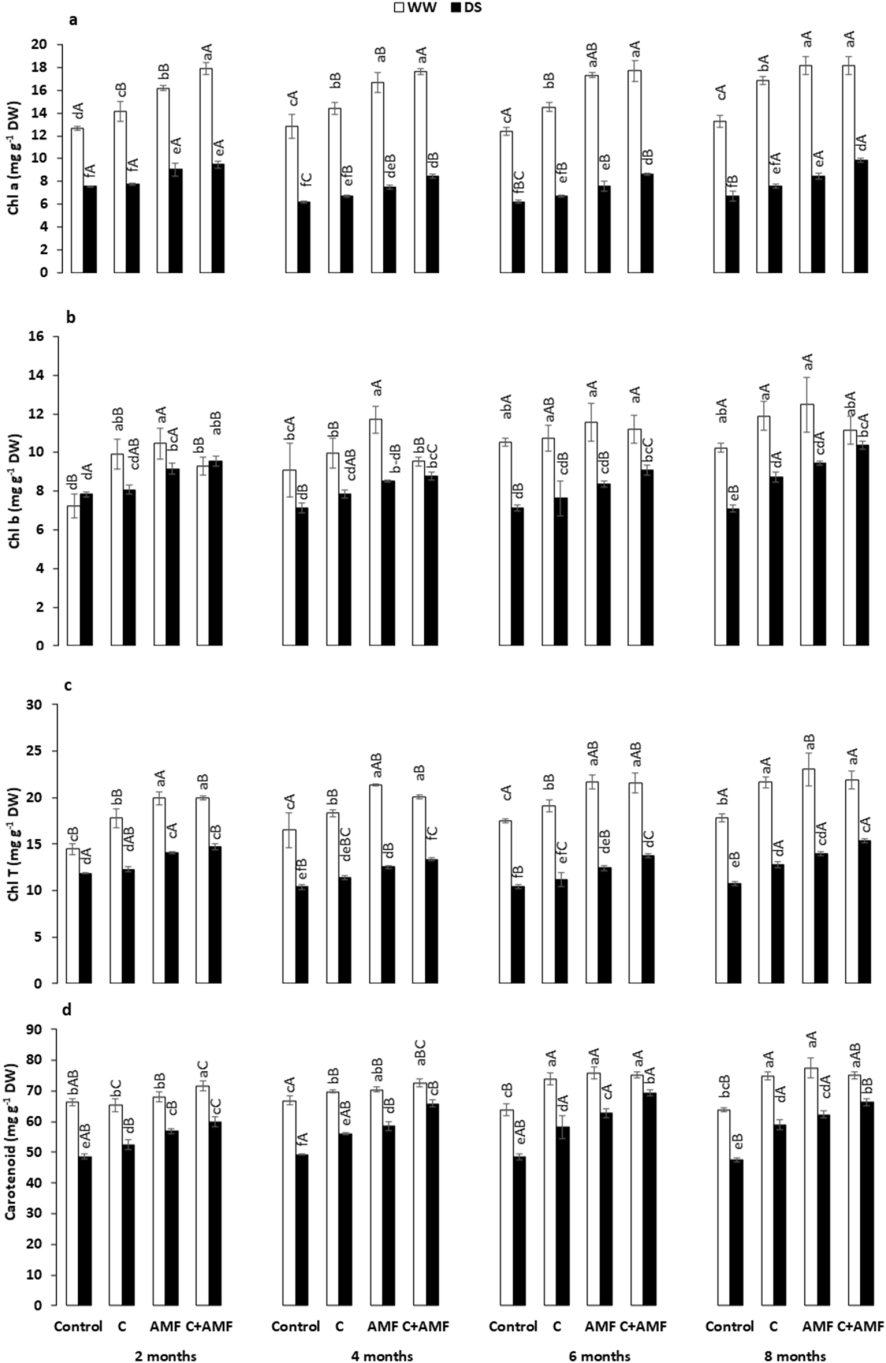
Effects of water regimes (WW: well-watered; open bars and DS: drought stress; filled bars) on (**a**) chlorophyll-a; chl ‘a’, (**b**) chlorophyll-b; chl ‘b’, (**c**) total chlorophyll; chl ‘T’, and (**d**) carotenoid in control carob plants and plants amended with compost (C) and/or assemblage of indigenous arbuscular mycorrhizal fungi (AMF). Data are mean ± SE (n = 5). Means followed by the same letters within the same time point are not significantly different at *P* < 0.05 (Tukey’s HSD). The upper case represents the differences within WW or DS water regime, and the lower case represents the differences within a one-time point.

Photosynthetic pigments concentrations decreased significantly (*P* < 0.001) after drought imposition, independently of the presence/absence of biofertilizers and timing (Fig. 4, Table 1). Under WW, the applied preparations increased the total chlorophyll and carotenoids concentrations in carob leaves, compared with the controls (Fig. 4c and d). The applied biofertilizers (AMF alone and the combination C + AMF) also increased the Chl “a”, “b”, total chlorophylls, and carotenoids content compared to non-inoculated and non-amended plants during an early stage in the first 2 months of the study and the following months throughout the year (Fig. 4a-d). Under DS, the highest level of pigments was yielded in plants treated with C + AMF, where Chl “a” (25, 37, 39 and 47%), “b” (22, 23, 46 and 46%), Chl “T” (24, 28, 32 and 43%), and carotenoids (23, 34, 43, and 45%) concentrations showed an upward trend after 2, 4, 6, and 8 months, respectively. Under DS conditions, these physiological attributes presented no significant difference over time for each treatment except C + AMF, which recorded a high improvement for all these traits, and control treatment showed a significant decrease for photosynthetic pigments throughout the 2-, 4-, 6-, and 8-months. The interaction among AMF, compost, and drought had a significant effect on Chl “a”, “b”, and total Chl (*P* < 0.001).

### Total soluble sugar and protein content

**Figure 5:**
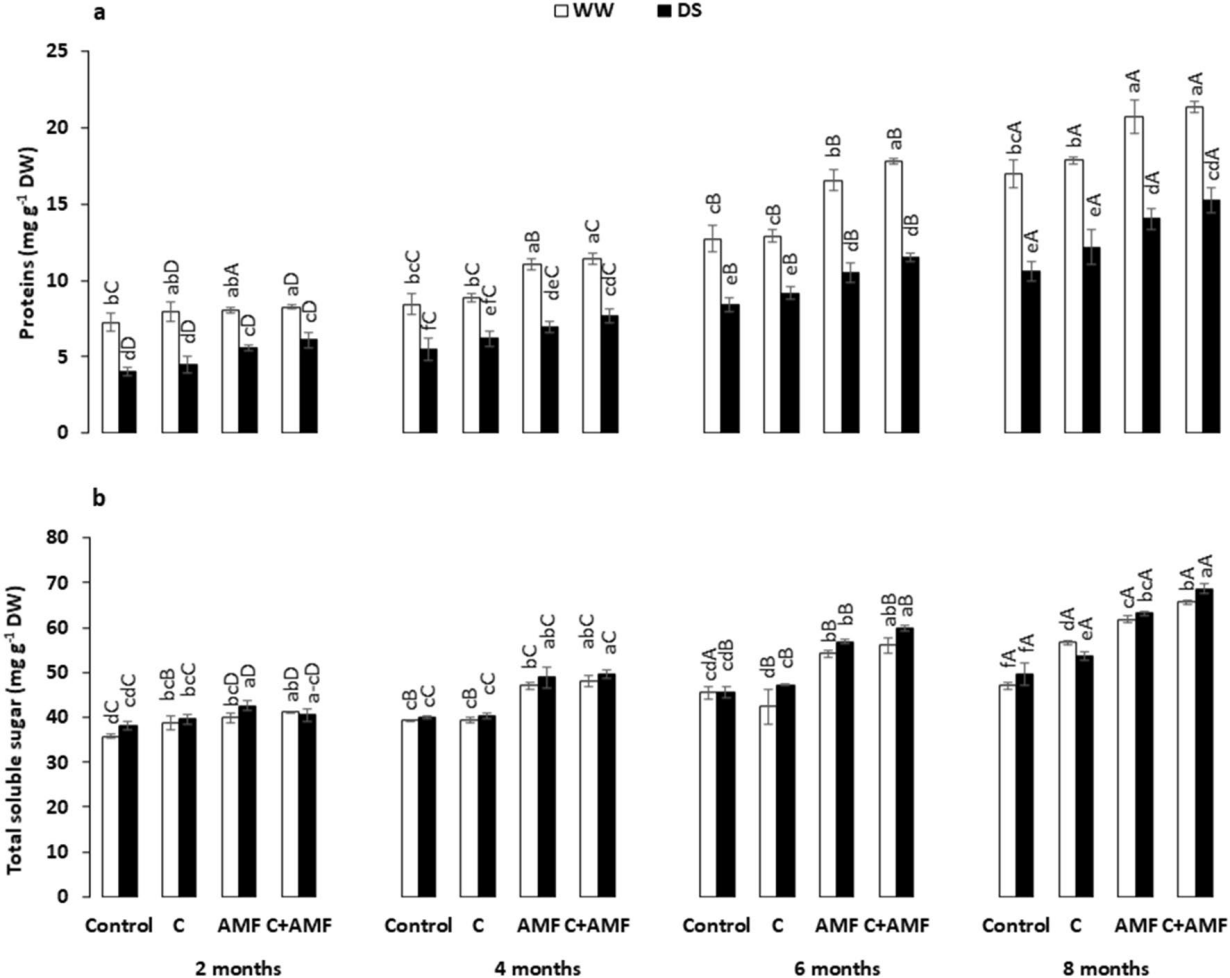
Influence of water regimes (WW: well-watered; open bars and DS: drought stress; filled bars) on (**a**) proteins and (**b**) total soluble sugars in control carob plants and plants amended with compost (C) and/or assemblage of indigenous arbuscular mycorrhizal fungi (AMF). Data are mean ± SE (n = 5). Means followed by the same letters within the same time point are not significantly different at *P* < 0.05 (Tukey’s HSD). The upper case represents the differences within WW or DS water regime, and the lower case represents the differences within a one-time point.

Under WW conditions, there was no effect of compost on the protein concentrations (Fig. 5a). The application of either AMF and the combination C + AMF treatments increased leaf protein concentrations under both WW—evident from month 4— and DS—from month 2—. The highest values of this parameter under water stress recorded in C + AMF reached ca. 50, 40, 36 and 45%, than respectively untreated plants after 2, 4, 6 and 8 months. The same trend of the protein concentration was observed in the TSS (Fig. 5b), which was increased by AMF and C + AMF applications. The highest amount of TSS was ca. 6, 25, 32, and 40% relative to the control plants after 2, 4, 6 and 8 months. Moreover and for each treatment, these parameters were significantly improved during the experiment period. The interaction among AMF, compost, drought, and timing were significant on TSS (*P* < 0.001, Table 1).

### MDA and H_2_O_2_ content and membrane stability

**Figure 6:**
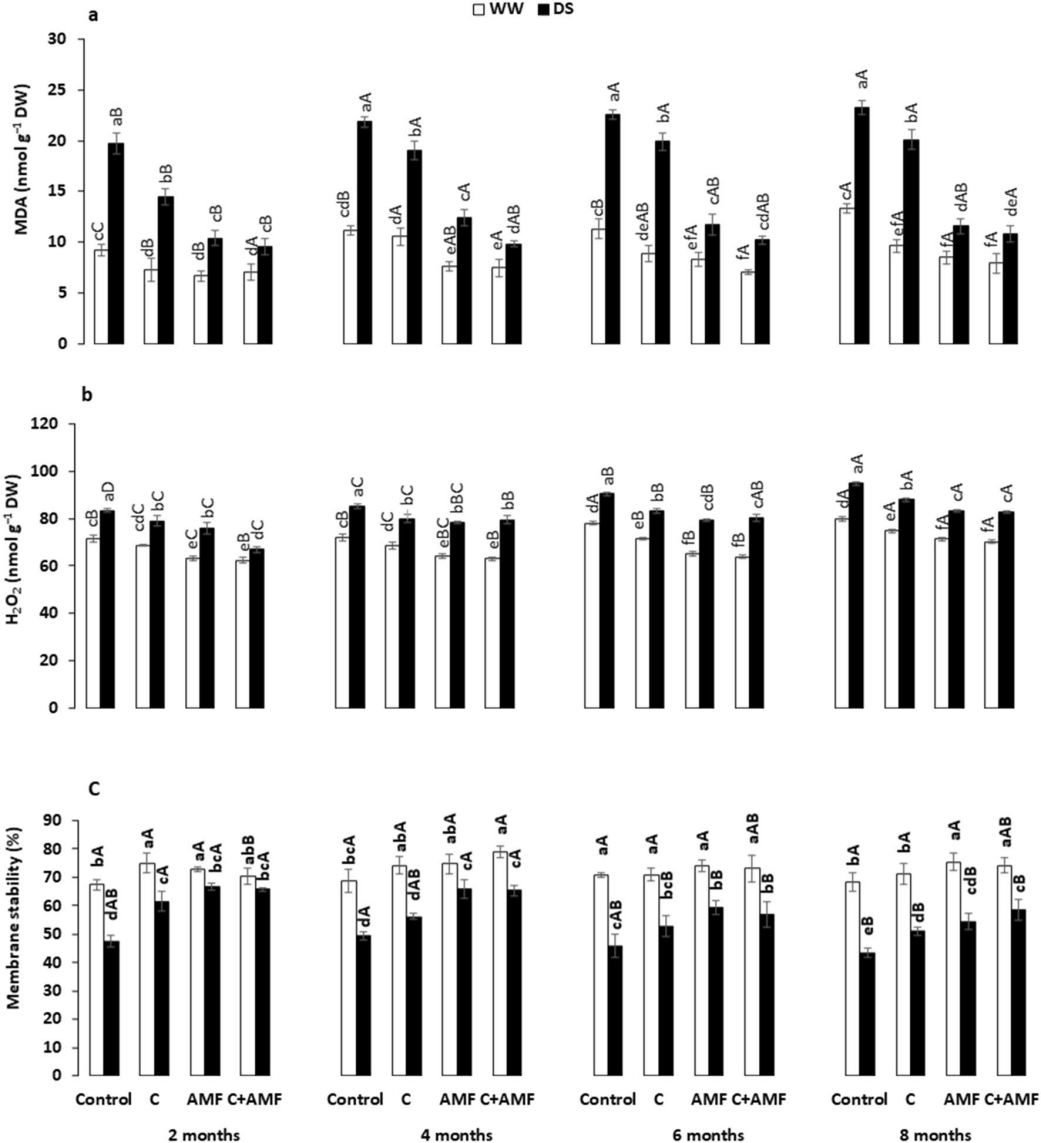
Effects of water regimes (WW: well-watered; open bars and DS: drought stress; filled bars) on (**a**) malondialdehyde; MDA, (**b**) hydrogen peroxide; H_2_O_2_ and (**c**) membrane stability in control carob plants and plants amended with compost (C) and/or assemblage of indigenous arbuscular mycorrhizal fungi (AMF). Data are mean ± SE (n = 5). Means followed by the same letters within the same time point are not significantly different at *P* < 0.05 (Tukey’s HSD). The upper case represents the differences within WW or DS water regime, and the lower case represents the differences within a one-time point.

Hydrogen peroxide (H_2_O_2_) and malondialdehyde (MDA) accumulation, and membrane stability (MS) are indicators of oxidative stress in plant tissues (Fig. 6). As presented in Fig. 6a and b, both MDA and H_2_O_2_ concentration significantly increased (*P* < 0.05) under DS compared to WW regime conditions. Under WW, membrane integrity values exceeded 67% regardless of the applied treatment during all the experiment periods (Fig. 6c). Under DS, in contrast, the membrane integrity steeply decreased below 50% in non-inoculated plants (notably in control plants) compared to AMF-inoculated plants and plants treated with C + AMF, showing values > 56% throughout the stress periods (Fig. 6c). H_2_O_2_ and MDA content increased significantly under DS conditions in control plants throughout the 2-, 4-, 6-, and 8-months. Upon applying AMF and/or compost, the H_2_O_2_ and MDA content decreased in the leaves under DS.

The lowest concentrations of MDA were observed in the leaves of treated plants with AMF and/or compost, these values being halved in C + AMF plants than control throughout 8 months’ stress. The interaction among AMF, drought, and timing significantly affected MDA and H_2_O_2_ (*P* < 0.001; Table 1).

### Antioxidant enzymes content

**Figure 7:**
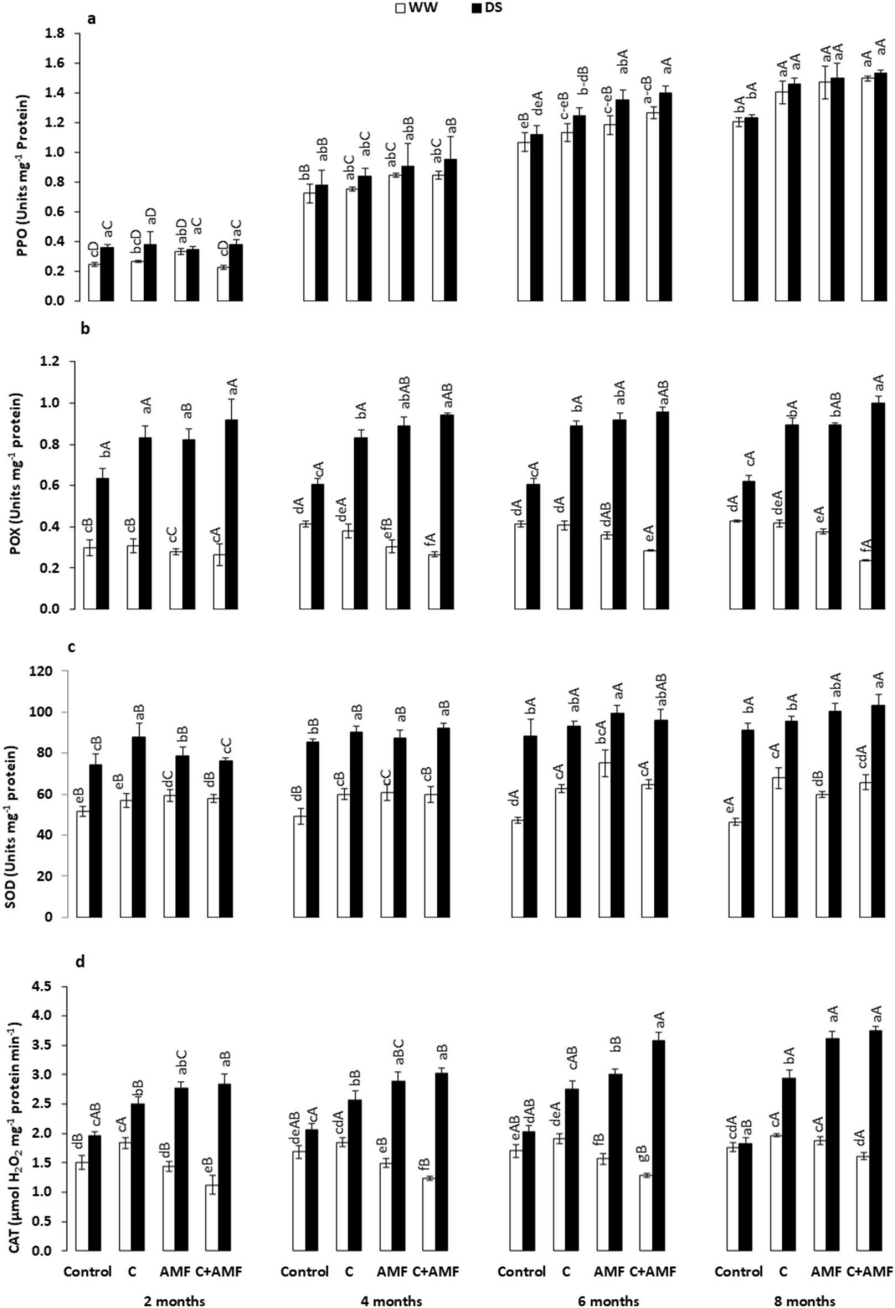
Effects of water regimes (WW: well-watered; open bars and DS: drought (**a**) polyphenoloxidase (PPO), (**b**) peroxidase (POX), (**c**) superoxide dismutase (SOD), and (**d**) catalase (CAT) activity in control carob plants and plants amended with compost (C) and/or assemblage of indigenous arbuscular mycorrhizal fungi (AMF). Data are mean ± SE (n = 5). Means followed by the same letters within the same time point are not significantly different at *P* < 0.05 (Tukey’s HSD). The upper case represents the differences within WW or DS water regime, and the lower case represents the differences within a one-time point.

Antioxidant enzymes are essential for scavenging ROS and for promoting plants resistance to droughts stresses. The present study studied the antioxidant enzymes activities on young carob plants under short- and long-term droughts conditions (Fig. 7). The obtained results showed that the activities of antioxidant enzymes (SOD, CAT, POX, and PPO) were significantly increased by biofertilizer treatments under water stress conditions. Under DS, the highest level of antioxidant enzymes was yielded in plants treated with C + AMF, where SOD (3, 8, 9 and 13%), CAT (45, 46, 76 and 104%), PPO (5, 23, 25 and 25%), and POX (45, 56, 58, and 61%) concentration showed an upward trend after 2, 4, 6, and 8 months, respectively. The significant increase in enzymatic antioxidants by applying AMF combined with compost suggested the role of these biofertilizers in young carob responses and their involvement in reducing oxidative damage induced by water stress over time. DS-treated plants showed a significant increase for each treatment over time, except POX values found not significant.

### Principal Component Analysis (PCA) and Heat mapping for growth, physiological, and biochemical traits of carob

**Figure 8:**
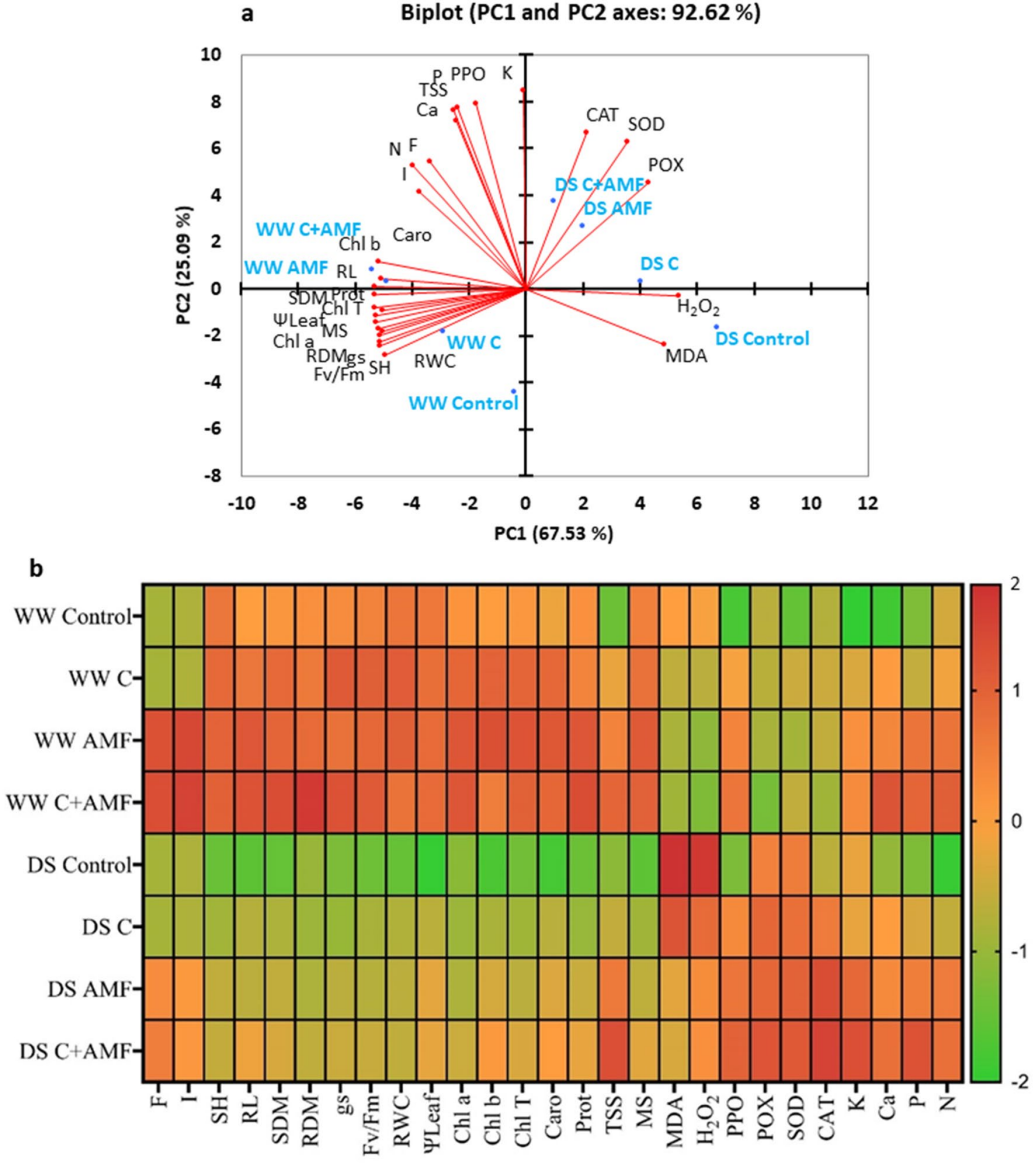
(**a**) Principal component and (**b**) Heat map analysis of the studied traits in young carob grown under WW (well-watered; open bars) and DS (drought conditions; filled bars) and treated or not (control) with AMF an/or compost. AMF: arbuscular mycorrhizal fungi; C: compost; F: frequency of mycorrhization; I: intensity of mycorrhization; SH: shoot height; RL: root length; SDM: shoot dry matter; RDM: root dry matter; ΨLeaf: Leaf water potential; RWC: relative water content; gs: stomatal conductance; Fv/Fm: chlorophyll fluorescence; Chl a: chlorophyll a; Chl b: chlorophyll b; Chl T: total chlorophyll; Car: Carotenoid; TSS: total soluble sugar; Prot: Protein; MS: membrane stability; H_2_O_2_: hydrogen peroxide; MDA: malondialdehyde; N: nitrogen; P: phosphorus; K: potassium; Ca: calcium; SOD: superoxide dismutase activity; CAT: catalase activity; POX: peroxidase activity and PPO: polyphenol oxidase activity.

Principal component analysis (PCA) considered all the analyzed parameters of the different treatments under both water regimes. Two main components accounted for 92.6% of the variability observed in the data, with 67.5% for PC1 and 25.1% for PC2 (Fig. 8a). Under the WW condition, PCA analysis showed a positive correlation among the AMF and/or compost for growth, physiological and water parameters, photosynthetic pigments, and protein content, which were highly correlated to each other. The analysis also confirmed the negative impact of DS conditions on these parameters. The biplot revealed the positive correlation linking control DS treatment and MDA and H_2_O_2_ content. Under DS conditions, PCA showed a clear positive correlation between the antioxidant system (SOD, CAT, and POX) and osmolytes (P, N, K, Ca and TSS) and the AMF application alone or combined with compost.

The heat map indicated the relative performance of the treated plants in both well-watered and water stress conditions. According to the color scale, the green darker color represents the highest values, while the darker red bands represent the lower values of carob seedlings under both conditions (Fig. 8b). SH, RL, SDM and RDM values showed moderate trends under DS conditions in the inoculated and composted plants compared to the control plants under normal conditions. Physiological parameters (gs, F_v_/F_m_, Chl a, b, T and carotenoids) showed a considerable variation between C + AMF carob seedlings under normal and stress conditions, but no significant change was found between C + AMF plants under DS and control plants under WW conditions. Under DS, leaf water potential and stress markers (MDA and H_2_O_2_) showed a contrasting effect under stress conditions in the control plants, as they had higher values than C + AMF carob seedlings under water stress conditions. In addition, enzyme activities (SOD, CAT, PPO, and POX) showed the highest intensities with increasing water deficit and were remarkably high in C + AMF carob plants than controls, showing thereby their direct involvement in the drought stress response mechanisms. The organic (proteins and sugars) and non-organic (P, N, Ca, and N) osmolytes increased following the stress conditions. This indicates that the osmolyte content depends mainly on the growing conditions (presence/absence of stress and/or biofertilizers), and this stress increases the accumulation of osmolytes in the leaves, as shown in Fig. 8b.

## Discussion

We investigated *herein* the effect of beneficial AMF and compost on growth, mineral uptake, physiology, and metabolites and ROS accumulation of *Ceratonia siliqua* cultivated under WW and DS conditions. The present study confirms AMF inoculation and compost addition effects on plant growth under water stress conditions. Furthermore, we suggest that the dual application of AMF and compost provides superior plant growth in the long term compared to AMF or compost applied separately. The improvement in growth and biomass accumulation when AMF and compost were applied in combination follows the previous studies^25,44,45^. The dual application of both biofertilizers improves plant growth and biomass accumulation, and water stress tolerance over time^44^. Bona et al*.*^46^ reported that inoculated strawberry plant by commercial AMF containing *R. intraradices*, *G. ageratum*, *G. viscosum*, *C. etunicatum*, and *C. claroideum* with organic amendment had higher growth than untreated plants. Likely, the improvement in plant growth observed with the dual application of AMF and compost may be due to the capacity of the AMF to acquire nutrients and water for the plants under DS conditions^47–49^. Besides, AMF symbiosis can increase the uptake of certain nutrients such as P and N, which directly benefits plant growth when organic fertilizers are used^50^. Phosphorus influences photosynthesis, biosynthesis of protein and phospholipids, membrane transport and cell division, which led to increased roots and shoots development, plant height, and dry biomass accumulation^51^. Phosphorous’s regulatory function in photosynthesis and carbohydrate metabolism of leaves can be considered one of the major factors limiting plant growth^52^. Previous studies have revealed the importance of using low doses of compost in combination with symbiotic microorganisms^48,53,54^. The addition of low dose compost, considered the optimal dose for plant growth with AMF, provides a reliable and predictable supply of nutrient “slow-release”, thereby allowing the AMF colonization of roots and stimulating the functioning of the complex compost-AMF, especially in soils with low nutrients^55–57^. These biostimulants (compost-AMF), applied individually or in combination, can improve crop production in less fertile soil systems. In contrast, an excessive application of compost amendment (≥ 20%) can hinder plant yield by, among other, altering/reducing microbial communities^58,59^.

The enrichment of soils low in organic matter by compost-derived substances provides many biological functions^60^. It can release and store nutrients and growth for plants, promote biological activity as a source of energy for microorganisms, and positively affect the structuring, physicochemical properties, and aeration of the soil^28,61^. As suggested by Roldán et al*.*^62^, the combined treatment of root mycorrhizal inoculation and soil amendment positively affect aggregate stability owing to the polysaccharides’ role. The greater uptake of nutrients led to enhanced root and shoot development, plant height, and dry matter accumulation under DS conditions^63,64^. It is interesting to note that compost and AMF had a generally synergetic effect on plant growth and AMF colonization under DS^25^.

In this study, no mycorrhizal structure was observed in the roots of non-inoculated carob seedlings, but the native mycorrhizal consortium successfully infected the AMF and C + AMF young carob. Several studies showed that root colonization decreased in the host plants under drought stress^65,66^. A short-term soil drought did not appear to discourage root colonization^20^, whereas a long-term soil drought considerably decreased root colonization and hyphal growth^67–69^. It is worth noting that the native soil composition and host plant genotype are also considered important in the recruitment of microbiome by the host plant. The functional profile of the microbial communities might be correlated with changes in community composition at the species level, suggesting that ‘suitability phenomena’ between the host plant and its native microbial communities, rather than a “generalistic” inoculum, may be functionally driven rather than driven by phylogeny^70^. Previous studies reported the effectiveness of native inoculum, which promotes and maintains plant growth despite the low colonization percentages under abiotic stresses^22,26,57^. Essahibi et al*.*^72^ reported that long-term drought stress decrease root colonization in carob seedlings inoculated with *Rhizophagus intraradices, R. fasciculatus* and *Funneliformis mosseae*. Furthermore, several studies showed that environmental conditions such as soil nutrition, moisture, temperature, and host genotype and age directly influence root and leaf microbial communities^73–79^. Our results showed that the application of green waste compost in sandy soil favored the spore production of the native AMF consortium, thereby improving the infectious propagules of carob plants. This may be related to the inherent capacity of the used consortium to sporulate or favorable interaction, due to C availability, with the carob host plant. Chaiyasen et al*.*^78^ showed that inoculation with indigenous AMF consisting of *Claroideoglomus etunicatum, C. etunicatum*, and *Funneliformis mosseae* in sandy soil amended with green waste compost containing 214 mg/Kg P improved spore numbers and the infectious propagule production in maize. Our results indicated that compost addition enhanced root colonization after long-term (after six months) compared to plants treated with AMF alone under DS. These results were consistent with previous studies reporting that compost and/organic amendment addition most often positively affected AMF growth under DS^25,78,80^. Several research had confirmed that water status in dry soil strongly altered AMF spore survival and germination and inhibited the spread of hyphae^67,81,82^. AMF exclusively found in the native habitat of wild relatives of crop plants comprise representatives of *Glomus*, *Rhizophagus*, *Funneliformis*, *Acaulospora*, *Entrophospora*, *Gigaspora,* and *Scutellospora*. The genus *Glomus* and *Glomus* related genera are known for its maximum germination^82^, and greater absorption of minerals and water under soil drying conditions^66^ and its capabilities to produce bioactive compounds (e.g. lysophosphatidylcholine) eliciting overexpression of fatty acid biosynthesis genes involved in the enhancement of AMF-host plant interactions^83^. Species including *R. intraradices*, *F. mosseae*, *R. clarus*, *R. diaphanum* can be found as indigenous soil inhabitants. Their exclusive presence in the native soil may be associated with their ability to improve the expression levels of nutrient transporter genes in fungal absorptive hyphae to increase mineral nutrients availability and translocation to the host plant^84^, thereby contributing to nutrient cycling in the undisturbed native soils^85^. These characteristics may ultimately affect seedling emergence, root development, and plant performance under (un) favorable conditions^86^. AMF facilitate seedlings establishment by enabling them to get access to limiting nutrients^87^. The diversity and abundance of *Glomus* species in soil, and their diversity in metabolic traits, makes *Glomeraceae* a potentially important family in soil nutrient cycling^88,89^. The genus *Acaulospora*, which belongs to the phylum *Acaulosporaceae*, encompasses strictly aerobic chemo-organotrophs that are adapted to acidic conditions^90^. *Acaulospora* members are generally known to produce a lot of mycelium but were slow to colonize the host plants likely because they mainly colonized from spores, as did the members of the *Gigasporaceae*^91^. Several studies indicate that extraradical mycelium (ERM) is highly infective in the *Glomeraceae* and *Acaulosporaceae* whereas members of the *Gigasporaceae* regenerate mostly from spores^92^. *Gigasporaceae* can live and reproduce with a small portion of C available resources^93^. *Scutellospora* and *Gigaspora*—have thick and strong hyphae—were likely able to colonize a new substrate area by themselves without the support of adjacent hyphae^94^. The healing process in these two fungi is presented as the most probable means of maintaining the viability of the hyphae in adverse conditions^95,96^. These AMF genera, when re-introduced into forestry soils, were able to establish and survive in the rhizosphere of carob and provided additional life-support functions (growth, tolerance) for the plants under DS. The organic amendments increased the mycorrhizal root colonization and hyphal length in soil^62^. The compost is rich in humic acid, which was reported to stimulate AMF hyphal network^97^. Medina et al*.*^98^ showed an increase in hyphal length and AMF activity grown in compost-free soil. In contrast, low root colonization of plants grown in soils non-amended with compost owing to the lack of suitable organic substrates^25^. This limitation may be due to reduced mycorrhizal hyphal growth, spore germination, and carbohydrate supplement from host plants under DS^99^. In the present study, compost maintained moderate levels of P, N, K, and water status. Therefore, the improvement in root colonization in the combination of compost and AMF is owing to the beneficial effects of compost on hyphae growth and spore germination under stressful conditions^80^. Besides, the positive effect of compost on plant growth and photosynthesis, thus possibly increasing carbohydrates transfer to AMF, indirectly improves mycorrhizal growth in roots. The dynamics of root colonization in the present study showed low mycorrhizal infection in the carob roots at early growth period (2 months after drought application) than late growth period (8 months after drought application). The AM fungal spore composition, therefore, might be affected by the altered soil, as well as the root mycorrhizal fungal groups, because of plant age or the need to beneficial partners in response to stresses and environmental stimuli. In particular, pioneering studies suggest that plants adopt the “cry-for-help” strategy by changing their root exudation chemistry to recruit beneficial microorganisms to resist (a)biotic stresses^100,101^. Other works on root colonization carried out on trees elucidated mechanisms of the establishment of the tree root microbiome and of tree root selection. Recently, Fracchia et al*.*^102^, indicated that the composition of the final root microbiome in host trees *Populus* relies on a series of colonization stages characterized by the dominance of different fungal guilds and bacterial community members over time. Pepe et al*.*^103^ reported on a time-course basis study that AMF life cycle in the soil, where fungal ERM, growing from both living and dead roots, represents a long-term survival structure functional to the prompt establishment of mycorrhizal symbioses and to the maintenance of soil biological fertility. Also, their data revealed that viability and functionality of certain AMF including *F. mosseae* and *R. irregulare* extraradical hyphae were uncoupled from host plant lifespan^103^. The overall growth dynamic of the microbiome, correlations between the soil properties and the spore densities, as well as the soil properties and root colonizations of chronosequence tree developing remain to be elucidated. Marupakula et al*.*^104^ revealed that the full microbiome is likely subjected to a complex dynamic during the colonization process of the roots of pines. The presence of compost, spore germination and mycorrhizal establishment, and plant photosynthesis and root growth, which favored mycorrhizal infection in the roots of older carob seedlings than in the younger ones. Previous studies have shown that the dynamics of mycorrhizal colonization of plant roots is related to soil properties and spore density and varies with plant species, mycorrhizal structures, AMF communities, period of growth, and seasonal conditions^105,106^.

Under DS, enhanced uptake nutrients are generally regarded as the essential benefit that AMF provide to their host plant^107^. Moreover, compost is a commonly used organic amendment to increase some nutrients and water storage capacity^47,108^. The dual application of compost and AMF has been suggested to ensure high uptake nutrients for plants^48,58^, especially under abiotic constraints such as water deficit^62^. In the present study, the AMF-compost combination application allowed a high P, K, N, and Ca uptake in the stressed plants than the control under WW conditions. This result corroborates Caravaca et al*.*^96^ and Zou et al*.*^109^, indicating that the nutrient uptake is more critical in inoculated plants under DS than WW conditions.

Furthermore, the amended soil with compost increased the leaf nutrient contents over the long term in mycorrhizal carob under DS than WW conditions. Ngo and Cavagnaro^110^ reported that applying organic amendment significantly increases the contents of available nutrients in the soil. Kohler et al*.*^53^ indicated that the synergistic relationships between AMF and compost affect the micronutrient elements' mobility in the rhizosphere and improve their bioavailability. Compost can improve soil productivity by increasing water and nutrient status for some limiting nutrients^25,47^. Compost is an excellent biofertilizer due to its high concentrations of significant macro- (e.g. N, P and K) and micro-nutrients^111^. Since compost is rich in organic and carbon matter, the transformation of these elements through soil microorganisms' mineralization process could release mineral nutrients in the soil. Moreover, AMF can also help increase the nutrients uptake by external mycelium via establishing an underground network that links the different plants and allows the transfer of these nutrients among them^63,112^. Under drought stress, the hyphal network still helps host plants sustain greater nutritional uptake^67^. Nutrient limitations have been overcome after AMF and/or compost addition, which helps the carob tree mitigate the adverse effects of droughts^25^.

In our study, C + AMF carob seedlings possessed significantly higher water status (leaf water potential and relative water content) under both WW and DS conditions, and the combination C + AMF was considerably higher, among the other treatments, under severe DS than WW conditions. These results were in line with improving the root colonization under WW and DS conditions. Wu et al*.*^67^ reported that mycorrhizal hyphae are essential in uptaking and transporting water from the soil to the host plant under DS conditions. However, AMF hyphae are likely to replace aquaporin activity in inoculated plants under water stress conditions^113^. Zou et al*.*^68^ revealed that the root aquaporins showed no changes or down-regulation under DS conditions. Mycorrhization has also improved soil water efficiency, contributing to better water and nutrient absorption to the host plant^114^. Li et al*.*^115^ have shown that organic amendments enhance soil water holding capacity, porosity, and water infiltration rate. In our study, the water status and nutrient content were more critical in plants grown under C + AMF treatment, particularly under long-term stress conditions. These results can be related to the AMF and compost roles in improving the nutrient and water status of the plant under DS. Previous studies demonstrated that the increase in water uptake by AMF resulted from nutrient increase via soil organic amendment^26,116^. The results obtained are consistent with those of Ortuño et al*.*^80^, suggesting that the combination of C + AMF improves water absorption under DS conditions. Moreover, C + AMF provides a higher root hydraulic conductivity contributing to the efficient water absorption by plants. The application of organic amendment and AMF (via hyphal networks) improves the formation of large soil macroaggregates, increasing the available volume of the soil solution under DS conditions^117^. Hashem et al*.*^116^ reported that the higher water uptake in C + AMF plants improves the water movement to evaporation surfaces resulting in increased stomatal opening.

Under DS conditions, the dehydration avoidance by C + AMF plants results from a balance between the capacity of water absorption, stomatal movements, and water distribution in plant tissues^118^. Haffani et al*.*^119^ showed that stomatal closure is another mechanism the plants use to reduce water loss to confront water stress. Our experimental conditions showed that in prolonged DS, stomatal conductance (g_s_) was significantly reduced in stressed plants. Under these conditions, the combination of AMF and compost contributed to more significant increases in g_s_. The higher g_s_ in carob trees can be due to several factors, such as increased hydraulic conductance^80^, increased root-fungi absorptive area, enhancing osmotic adjustment^80,118^, and/or improved mycorrhizal colonization^44^. Our data revealed that higher mycorrhizal colonization and osmotic adjustment in plants subject to C + AMF treatment helped improve the plant's water status, as demonstrated by the less negative Ψ_Leaf_ values. Plants grown in the presence of AMF and compost often show higher g_s_ under water stress over time^80,120^. This improvement of physiological traits (higher water status and stomatal opening) in plants amended with compost and inoculated by AMF can lead to an increase in efficiency of photosystem II (PSII) (F_v_/F_m_) under DS. In this study, the combination of AMF with compost increased F_v_/F_m_ in plants exposed to long-term water stress conditions compared to the control. This result demonstrates that the application of AMF can maintain a normal utilization of light energy in plants photochemical processes under DS. Similarly, some studies have demonstrated AMF inoculation's capacity to increase the maximum quantum yield of PSII under DS^20,24,121^. Gong et al*.*^122^ have reported that two *Glomus* species significantly increased the F_v_/F_m_ in *S. davidii* compared to non-inoculated plants under DS conditions. AMF inoculation under DS improved the energy cycling between the reaction center and the chloroplast pool, and it enhanced the efficiency of excitation energy capture by chloroplasts and decreased the injury caused to the photosystem reaction centres^123,124^.

DS has been reported to affect the photosynthetic apparatus's pigment composition and varies across species due to adaptation to different environments^4,125^. Differences in short- and long-term pigment pool size responses might contribute to intraspecific variation in photosynthetic C assimilation and photoprotective mechanisms under drought^126^. Our data showed that the AMF-Compost combination protects the chlorophyll and carotenoids against degradation. The application of compost in soil significantly improved the chlorophyll content in plants under DS conditions^127,128^. Similarly, the role of AMF to reduce the negative effect of DS by improving chlorophyll and carotenoid content has been demonstrated^2,129^. Therefore, the beneficial effect of AMF-compost application may be related to compost in improving root colonization. Osmolytes and mineral content increases may, at least partly, mediate the overall increase in chlorophyll content under DS of non-inoculated plants growing in amended soil. Nutrient concentrations were higher in C + AMF treatment than in control, regardless of the stress length and water regime conditions. High water potential to open stomata results in increased C assimilation and higher chlorophyll content under DS^40^. The beneficial effect of these biofertilizers on plant physiology is not related only to better acquisition of water and nutrients, photosynthesis and chlorophyll content but also to increased carbohydrates and proteins^130^.

Several studies have reported a positive effect of carbohydrates in regulating the osmotic potential of cells which, in turn, improves water absorption under unfavorable condition^131,132^. Our data indicated that the concentrations of soluble sugars and protein in treated plants, particularly those amended with C + AMF, significantly increased during all periods of WW and DS. These results agree with several studies using AMF and/or compost under the same conditions^26,133^. AMF-inoculated carob accumulated more sugar content during the prolonged DS conditions, likely to maintain high hydration and turgor level, which maintain overall physiological activities under harsh environments^4,72^. Indeed, the carbohydrates accumulation in DS conditions reduced the osmotic potentials in host cells^40^. The leaf sugar content in inoculated carob was higher than non-inoculated plants, confirming Abbaspour et al*.*^40^ findings suggesting that the natural physiological metabolism of non-inoculated plants was less active than amended plants during WW and DS conditions. Under DS, the higher content of soluble proteins in mycorrhizal plants grown in soil amended with compost may explain the strengthening of the non-enzymatic antioxidant defense system by AMF^66^.

Under DS, the membrane lipids are highly susceptible to reactive oxygen species (ROS) oxidation^134,135^. The ROS created in plants under DS breaks up lipid membranes, producing MDA, and decreasing membrane stability (MS) in *Ceratonia siliqua* L cells^136^. In the present study, the DS elevated MDA content and reduced MS, while AMF inoculation decreased MDA generation and increased carob MS, especially in amended soils throughout the whole year experiment. These results suggest that the C + AMF combination may suppress the high intracellular H_2_O_2_ levels and have a vital protective role in reducing cell membrane damage (increase in MS) under DS. Similar results were observed by Duo et al*.*^26^ in *Festuca arundinacea*. Abbaspour et al*.*^40^ showed a positive correlation between the low H_2_O_2_ and MDA content and water stress tolerance in amended and inoculated plants.

The drought tolerance of carob has been attributed to the induction of the antioxidant enzyme system. The present study showed that water stress significantly increased antioxidant enzymes (SOD, CAT, POX, and PPO) in the carob seedlings over the time. This activity was increased more significantly in treated plants, particularly those grown with the combination of AMF and compost under DS. Similar results were reported previously for different species^24,133^. In addition, Anli et al*.*^24^ showed a significant increase of POX and PPO in plants inoculated with AMF and/or amended with compost than controls under DS. Dumanović et al*.*^137^ reported that greater antioxidant activities favour the removal of reactive oxygen species (ROS), which leads to greater drought resistance in plants. Kirova et al*.*^138^ and Torun^139^ demonstrated that SOD and POX activities were increased in plants and related to protection against oxidative damage due to DS. The SOD enzyme transforms superoxide into hydrogen peroxide (H_2_O_2_), which is then converted to O_2_ and water by the antioxidant enzymes CAT and POX^140^. Under DS, the activities of SOD and CAT can prevent or decrease the formation of this oxide^141^. Our results showed that the carob seedlings inoculated and/or amended with compost increased antioxidant activities (SOD, CAT, PPO, and POX) over the DS period drought, consistent with the increment in peroxidation levels^141^. Several studies revealed that compost alone or combined with AMF minimized the effects of abiotic stress by increasing antioxidant enzymes and decreasing MDA and H_2_O_2_^75^. A recent study^72^ reported that the inoculation of carob seedlings with three AMF species, namely *Rhizophagus intraradices, R. fasciculatus,* and *Funneliformis mosseae* promoted its capacity to tolerate water stress by improving water and nutrients uptake, stomatal conductance, membrane stability, osmotic adjustment and antioxidant system. On the other hand, Jadrane et al*.*^136^ suggested that AMF inoculum applied at 2 g of mycorrhized root fragments can improve carob drought resistance by improving cell membrane stability and elasticity by activating the antioxidant defense system.

Overall, our results revealed that the carob tree drought tolerance mechanism is progressively improved over time in treated plants, particularly plants grown in AMF and compost. The carob growth has been increased by promoting the physiological traits, nutrient contents, antioxidant defense system, and a decrease in stress markers (H_2_O_2_ and MDA) in C + AMF plants, resulting in significant protection of the photosynthetic machinery membrane stability under DS conditions.

## Conclusions

Water deficits drastically impact carob growth, affecting biomass production, mineral nutrient acquisition, water status, photosynthetic pigment content, and cell membrane stability. Under DS conditions, the application of indigenous AMF with green-made compost can reduce water stress's detrimental effects. The applied biofertilizers mitigated the water stress-induced changes by improving the antioxidant defence system, thus preserving cells components from oxidative damage. The mechanisms of action for these biofertilizers in maintaining physiological functions are probably related to improved water relations, mediation of photosynthesis and ionic homeostasis parameters, higher membrane stability, and lower lipid peroxidation under water deficit. At the cellular level, biofertilizers-mediated drought tolerance allowed biochemical protection measures to be activated in carob seedlings in response to drought to avoid the negative consequences of stress-induced ROS and to withstand a prolonged period of water stress. Hence, the management of natural resources as AMF and compost assured plant growth and establishment under prolonged water limitation, providing a valuable tool for the recovery of degraded forest.

## Materials and methods

### Biological material and application of biofertilizers

Scarified carob seeds with sulfuric acid were germinated in Petri dishes on moist filter paper and were incubated at 28 °C for five days and then rinsed and immersed in sterile distilled water for 24 h. The uniform-looking seedlings were transplanted, one plant per pot, in 5 kg plastic pots filled with sterilized forestry soil (at 0.11 MPa and 121 °C for 2 h), containing 1% organic matter, 0.6% total organic carbon (TOC), 8 mg/Kg available P, 900 mg/Kg N, 2356 mg/Kg Ca, and 568 mg/g available K, 8.2 pH, and 0.14 mS/cm electrical conductivity. The soil texture used was: 51% sand, 19% clay, and 30% loam.

We extracted and isolated the native AMF community comprising AMF spores from the rhizospheric soil field of five different regions in Morocco (Taounate, Taza, Errachidia, Zagora, and Essaouira). All spores were extracted from the respective inoculum using the wet sieving and sucrose centrifugation method^142^ and were examined ad libitum to confirm AMF identity and viability as per Souza^143^ and Kachkouch et al*.*^144^. The number of AMF spores detected in this inoculum was 165 spores/100 g of the soil sample. The inoculum contains a mixture of 26 species classified into 8 genera Glomeromycota (*Glomus*, *Rhizophagus*, *Funneliformis*, *Acaulospora*, *Entrophospora*, *Gigaspora*, *Claroideoglomus*, and *Scutellospora*) and 6 families (Glomeraceae, Acaulosporaceae, Entrophosporaceae, Diversisporaceae, Claroideoglomeraceae and Gigasporaceae ), and 2 orders (Glomerales et Diversisporales): *Rhizophagus proliferus* (= *Glomus proliferum*), *Claroideoglomus etunicatum* (= *Glomus etunicatum*), *Rhizophagus clarus*, *Rhizophagus diaphanus* (= *Glomus diaphanum*), *Rhizophagus intraradices*, *Funneliformis mosseae*, *F. geosporum*, *F. constrictum* (= *Glomus constrictum*), *Diversispora epigaea Glomus* sp. 1, *Glomus* sp. 2, *Glomus* sp. 3, *Glomus* sp. 4, *Glomus* sp. 5, *Acaulospora denticulata*, *A. spinosa*, *A. kentinensis*, *Acaulospora* sp. 1, *Acaulospora* sp. 2, *Acaulospora* sp. 3, *Acaulospora* sp. 4, *Entrophospora* sp. 1, *Gigaspora* sp. 1, *Gigaspora* sp. 2, *Gigaspora* sp. 3, and *Scutellospora* sp. 1.

We produced the compost locally from green waste as described previously by Meddich et al*.*^145^. The physicochemical and microbiological properties of the compost are 6.87 pH, 2.5 mg/g Olsen available P, 800 mg K /Kg, and 1900 mg Ca /Kg, 306.5 g C /kg organic, 551.7 g/kg organic matter, 21.9 g N /kg, 14 C/N ratio, and 490 g/kg ash content.

### Experimental design

The experiment consisted of four treatments: (i) Control: not inoculated and compost-free treatment, (ii) AMF: plants inoculated with 60 g of AMF inoculum containing about 100 spores and 2 g of mycorrhizal roots fragments (with high AMF infection frequency (96%) and intensity (60%)) containing hyphae, vesicles, and spores, (iii) C: plants treated with 175 g/plant of compost alone (5% *W*/*W* with respect to culture soil), and (iv) C + AMF: plants treated with a combination of AMF and compost. Young carobs were arranged in a fully randomized block design, and there were 30 biological replicates per treatment. To standardize the background non-AMF microbial community within each pot, all pots received microbial filtrate (300 mL) made of equal parts of extraneous extraction solution (i.e., without AM fungal spores) from the AMF consortium (including the forest soil before sterilization).

The young carobs were watered and maintained at 70% field capacity (FC) for four months. Soil moisture was measured for all pots using a TDR meter (Delta UK Ltd., Clacton-on Sea, UK) in the morning and evening of each day. The amount of needed water under different water conditions was calculated according to the measured soil water content, soil bulk density, soil moisture maximum field capacity, and soil weight. Then, young carobs were subjected to two water regimes: 70% FC (WW; well-watered) and 35% FC (DS; severe drought stress) conditions. Plants were maintained for 12 months in semi-controlled conditions with natural light (photon flux density ranged from 500 to 750 µmol m^−2^ s^−1^), average temperature 23 °C (day/night air temperatures of 28/18 ± 4 °C), and average relative humidity 70%. Pots were rearranged within the greenhouse every week to reduce any spatial effects, and plant material harvest was carried out every two months.

The design of the experiment was factorial with the following different factors:

Factor1: the application of AMF and/or compost, factor 2: the water stress (well-watered or severe stress conditions), and factor 3: treatments (AMF and/or compost and/or drought) timing (2, 4, 6, and 8 months).

The experimental research on carob plants, including the collection of plant material, has been conducted in accordance with local, national, and international guidelines and legislation.

### Plant harvest, mineral nutrient content, leaf water potential kinetics, and fungal colonization

After 2, 4, 6, and 8 months, fully expanded leaves of plants cultured in the absence or presence of biofertilizers and-or drought stress were harvested at the end of the light period, snap-frozen, and ground to a fine powder in liquid nitrogen with a pestle and mortar for the subsequent analyses. Carob seedlings' growth performance was assessed by measuring the shoot height, root length, and shoot and root dry matters (DM; obtained after drying samples at 80 °C until the weight remained constant). The dry shoot samples were then ground to a fine powder using a pulverising mill before measuring the total N, P, K, and Ca. Total N content was measured according to the method described by Jones^146^. Available P was determined according to Olsen and Sommers method^147^. The amount of K and Ca content was measured by flame spectrophotometer (AFP100) as described by Wolf (1982).

Leaf water potential (Ψ_Leaf_) was measured using a pressure chamber (Model 600-EXP Super Pressure Chamber, PMS instrument, Albany, OR, USA) at predawn (06:00–08:00 h). Measurements were performed on fully developed leaves from the upper part of the five plants' stemper treatment.

To quantify the mycorrhizal colonization in each harvest, root samples were carefully washed, cleared with 10% KOH at 90 °C for 2 h, acidified with 5% lactic acid for 20 min, and stained with 0.05% (w/v) Trypan blue for 30 min at 90 °C^148^. Total arbuscular and vesicular colonization were assessed using the gridline-intersect method with at least 100 intersects per sample^149^.

### Relative water content kinetics

Leaf relative water content (RWC%) was measured according to Barrs and Weatherley^150^. Leaf discs (1 cm^2^) were cut out from the third leaf of the apex (most expanded leaf), weighed to obtain fresh leaf weight (FW), and immediately hydrated by floating on deionized water to full turgidity for 24 h under darkness at 4 °C. Then, the samples were immediately weighted to determine the maximum turgidity (TW). Subsequently, these discs were dried at 70 °C until the weight was constant to determine the dry matter (DM). The leaf RWC was measured using the following formula:

$$RWC\left(\%\right)=\frac{FW-DM}{\mathrm{TW}-\mathrm{DM}}\times 100$$

### Stomatal conductance, chlorophyll fluorescence and photosynthetic pigments kinetics

Stomatal conductance (g_s_) was measured on mature leaves using a portable steady-state diffusion porometer (Leaf Porometer LP1989, Decagon Device, Inc., Washington, USA). Five measurements per treatment were made on the abaxial side per plant (between 09:30–11:00 a.m).

Chlorophyll fluorescence (F_v_/F_m_) was measured in dark-acclimated (using leaf clips for 30 min) youngest fully expanded leaves using a hand-held fluorometer (Opti-sciences OSI 30p, Hudson, NY, USA). The measured F_v_/F_m_ corresponded to the quantum yields (F_v_/F_m_ = (F_m_− F_0_)/F_m_), where F_m_ and F_0_ are the maximum and initial quantum yields of dark-adapted leaves, respectively.

The chlorophyll a, b, total chlorophyll, and carotenoid concentration was determined according to the method described by Arnon^151^. Photosynthetic pigments were extracted from the frozen leaf powder using 80% (v/v) cold acetone. The extracted material was centrifuged at 10,000 × *g* at 4 °C for 10 min, and the supernatant was retained. Supernatant absorbance was read at 480, 645, and 663 nm using a UV–visible spectrophotometer (UV-3100PC spectrophotometer, VWR).

### Total soluble sugars and proteins quantification kinetics

The total soluble sugar (TSS) content was measured in 0.1 g of frozen samples homogenized with 4 mL of ethanol (80%). The resulted supernatant was mixed with 0.25 mL of phenol (5%) and 1.25 mL of concentrated sulfuric acid. The absorbance was measured at 485 nm in a UV-3100PC spectrophotometer, according to Dubois et al*.*^152^. Protein concentration was determined by the Pierce 660 nm Protein Assay Kit (Thermo Fisher Scientific, Waltham, MA, USA) using bovine serum albumin (BSA) as a standard.

### Malondialdehyde and hydrogen peroxide content

Malondialdehyde (MDA) was determined according to Savicka and Škute^153^ method. In brief, lipid peroxides were extracted from leaves with 10 mL of 0.1% (w/v) trichloroacetic acid (TCA). After centrifugation (18,000 × *g* for 20 min), the chromogen was formed by mixing 1 mL of supernatant with 2.5 mL thiobarbituric acid (TBA). The mixture was incubated at 95 °C for 30 min, and the reaction stopped by placing the tubes in an ice bath. The chromogen formed was measured at 450, 532, and 600 nm.

MDA content was calculated as follows: $\left[MDA\right]=6.45\times \left(A5324A600\right)-0.565A450$

Hydrogen peroxide (H_2_O_2_) content in leaves was determined according to Velikova et al*.*^154^. The frozen leaf subsamples were mixed with 5 mL of 10% (w/v) TCA and then centrifuged for 15 min at 15,000 × *g* at 4 °C. The supernatant (0.5 mL) was recovered and then mixed with 0.5 mL of potassium phosphate buffer (10 mM, pH 7.0), and 1 mL of iodic potassium (1 M) was added. After 1 h of incubation in the dark at room temperature, the absorbance at 390 nm was recorded and plotted against a standard H_2_O_2_ curve.

### Determination of membrane stability kinetics

Membrane stability (MS%) was determined after each harvest by measuring the electrolyte leakage according to Shanahan et al*.*^155^. Briefly, leaf discs were washed with deionized water to remove any surface-adhered electrolytes. Samples were placed in individual stoppered vials containing 10 mL of distilled water and kept at 25 °C on a shaker (100 rpm) for 6 h. The initial electrical conductivity (EC1) was measured using a conductivity meter (Hannah Instruments HI8820N). Then, the leaf discs were placed in a thermostatic water bath at 95 °C for 15 min, and the second reading (EC2) was determined after cooling the solutions to 25 °C. The Membrane Stability (MS) was calculated as:

$$MS\left(\%\right)=\left[1-\left(\frac{EC1}{EC2}\right)\right]\times 100$$

### Determination of antioxidant enzymes kinetics

Fresh leaf (0.1 g) ground with liquid nitrogen was homogenized in a cold mortar with 4 mL of 1 M phosphate buffer (pH 7) and 5% polyvinylpolypyrrolidone. The mixture was centrifuged at 18,000 g for 15 min at 4 °C. Then, the supernatant obtained was used to measure the antioxidant enzyme activities^156^. Briefly, superoxide dismutase (SOD) activity was assayed by Beyer and Fridovich^157^ method. One unit of SOD activity was defined as the ability to inhibit 50% of the photochemical reduction of p-nitroblue-tetrazolium (NBT) at 25 °C. The SOD activity was expressed at unit min^−1^ mg protein^−1^. The catalase (CAT) activity was measured by monitoring the decrease in absorbance at 240 nm for 3 min following the consumption of H_2_O_2_ substrate at 240 nm for 3 min^158^. Peroxidase (POX) activity was measured for 3 min using the method described by Tejera-Garcıa et al*.* (2004). The reaction mixture was composed of K_2_HPO_4_/KH_2_PO_4_ (100 mM) buffer K_2_HPO_4_/KH_2_PO_4_ (pH 6.5), guaiacol (40 mM), H_2_O_2_ (10 mM) and enzyme extract (0.1 mL). The results obtained were expressed at units mg of protein^−1^. Polyphenoloxidase (PPO) activity was evaluated by monitoring the oxidation of catechols at 410 nm, according to Gauillard et al*.*^159^. The mixture used contains K_2_HPO_4_/KH_2_PO_4_ buffer (100 mM, pH 6), catechol (50 mM) and an enzyme extract. The activity of PPO was expressed at units mg of protein^−1^.

### Statistical analysis

Data are presented as mean ± SE (standard error) of five independent biological replicates. All results were subjected to a multifactorial analysis of variance (MANOVA) using SPSS 23.0 software (IBM, Armonk, NY, USA) for the tested factors (AMF; A, Compost; C, drought; D, and time of the four harvests; T) and their interactions. Mean comparisons were carried out using Tukey’s honest significant difference test using a significance level of 5% (p ≤ 0.05). Principal component analysis (PCA) was performed using XLSTAT-software v. 2016, and Heatmap was performed using software GraphPad® Prism v9.0 to categorize various growth, physiological, and biochemical characteristics of carob plants under the two water regimes (DS and WW) that illustrate the distinction between the variables and the different treatments applied.

## Supplementary Information

Supplementary Information.
