# Supplementary material for: Assemblage of indigenous arbuscular mycorrhizal fungi and green waste compost enhance drought stress tolerance in carob (Ceratonia siliqua L.) trees
Source: Sci Rep. 2021 Nov 24;11:22835. doi: 10.1038/s41598-021-02018-3 (PMC8613250; doi:10.1038/s41598-021-02018-3)
Supplement: Supplementary file 1 — Supplementary Information. [file 41598_2021_2018_MOESM1_ESM.doc]

**Supplementary Table 1** Loading values and percent contribution of variables on the axis identified by the principal component analysis for all treatments under drought and well-watered conditions

| Variable | PC1 | | PC2 |  | PC3 | |  | |  |
| --- | --- | --- | --- | --- | --- | --- | --- | --- | --- |
|  | Loading values | Contribution of variable (%) | Loading values | Contribution of variable (%) | Loading values | | Contribution of variable (%) | |  |
| gs | **-0.955** | 5.005 | -0.226 | 0.754 | | 0.019 | | 0.032 | |
| Fv/Fm | **-0.959** | 5.048 | -0.258 | 0.981 | | 0.069 | | 0.425 | |
| RWC | **-0.925** | 4.695 | -0.325 | 1.562 | | 0.176 | | 2.782 | |
| ΨLeaf | **-0.939** | 4.837 | -0.105 | 0.162 | | 0.289 | | 7.509 | |
| Chl a | **-0.969** | 5.151 | -0.193 | 0.552 | | -0.082 | | 0.596 | |
| Chl b | **-0.945** | 4.893 | 0.049 | 0.035 | | 0.248 | | 5.534 | |
| Chl T | **-0.981** | 5.279 | -0.130 | 0.251 | | 0.021 | | 0.038 | |
| Caro | **-0.967** | 5.126 | 0.132 | 0.257 | | 0.176 | | 2.787 | |
| Prot | **-0.992** | 5.400 | -0.030 | 0.013 | | -0.084 | | 0.629 | |
| TSS | -0.452 | 1.122 | **0.878** | 11.378 | | -0.021 | | 0.038 | |
| F | **-0.633** | 2.198 | **0.616** | 5.592 | | -0.385 | | 13.285 | |
| I | **-0.700** | 2.688 | 0.470 | 3.262 | | -0.483 | | 20.904 | |
| MS | **-0.979** | 5.259 | -0.163 | 0.393 | | 0.091 | | 0.736 | |
| MDA | **0.903** | 4.475 | -0.271 | 1.081 | | -0.220 | | 4.345 | |
| H2O2 | **0.996** | 5.447 | -0.032 | 0.015 | | -0.047 | | 0.195 | |
| PPO | -0.324 | 0.574 | 0.902 | 12.002 | | 0.077 | | 0.535 | |
| POX | **0.801** | 3.519 | **0.516** | 3.937 | | 0.280 | | 7.033 | |
| SOD | **0.664** | 2.420 | **0.717** | 7.592 | | 0.068 | | 0.412 | |
| CAT | 0.397 | 0.865 | **0.758** | 8.488 | | 0.493 | | 21.771 | |
| K | -0.016 | 0.001 | **0.962** | 13.657 | | -0.119 | | 1.273 | |
| Ca | -0.453 | 1.124 | **0.815** | 9.808 | | -0.117 | | 1.235 | |
| P | -0.472 | 1.224 | **0.868** | 11.115 | | -0.056 | | 0.278 | |
| N | **-0.743** | 3.029 | **0.596** | 5.249 | | 0.161 | | 2.318 | |
| SH | **-0.956** | 5.012 | -0.276 | 1.124 | | 0.089 | | 0.718 | |
| RL | **-0.994** | 5.414 | 0.010 | 0.001 | | -0.005 | | 0.003 | |
| SDM | **-0.989** | 5.363 | -0.091 | 0.123 | | 0.009 | | 0.007 | |
| RDM | **-0.939** | 4.833 | -0.204 | 0.616 | | -0.226 | | 4.583 | |
| Eigen value | 18.232 | | 6.775 | | | 1.116 | | | |
| Variability (%) | 67.525 | | 25.093 | | | 4.132 | | | |
| Cumulative % | 67.525 | | 92.618 | | | 96.750 | | | |

gs: stomatal conductance; Fv/Fm: chlorophyll fluorescence; RWC: relative water content; ΨLeaf: Leaf water potential; Chl a: chlorophyll a; Chl b: chlorophyll b; Chl T: total chlorophyll; Car: Carotenoid; Prot: Protein; TSS: total soluble sugar; F: frequency of mycorhization; I: intensity of mycorhization; MS: membrane stability; MDA: malondialdehyde; H2O2: hydrogen peroxide; PPO: polyphenol oxidase activity; POX: peroxidase activity SOD: superoxide dismutase activity; CAT: catalase activity; K: potassium; Ca: calcium; P: phosphorus; N: nitrogen; SH: shoot height; RL: root length; SDM: shoot dry matter and RDM: root dry matter. Values in bold explained >50% contribution to the significant component.
